# Charge and lipophilicity are required for effective block of the hair-cell mechano-electrical transducer channel by FM1-43 and its derivatives

**DOI:** 10.3389/fcell.2023.1247324

**Published:** 2023-10-10

**Authors:** Marco Derudas, Molly O’Reilly, Nerissa K. Kirkwood, Emma J. Kenyon, Sybil Grimsey, Siân R. Kitcher, Shawna Workman, James C. Bull, Simon E. Ward, Corné J. Kros, Guy P. Richardson

**Affiliations:** ^1^ Sussex Neuroscience, School of Life Sciences, University of Sussex, Brighton, United Kingdom; ^2^ Sussex Drug Discovery Centre, School of Life Sciences, University of Sussex, Brighton, United Kingdom; ^3^ Department of Experimental Cardiology, Academic Medical Center, Amsterdam, Netherlands; ^4^ School of Biosciences, University of Kent, Canterbury, United Kingdom; ^5^ School of Medicine, Institute of Life Sciences, Swansea University, Swansea, United Kingdom; ^6^ Section on Neuronal Circuitry, National Institute on Deafness and Other Communication Disorders NIH, Bethesda, MD, United States; ^7^ Department of Biosciences, College of Science, Swansea University, Swansea, United Kingdom; ^8^ Medicines Discovery Institute, Cardiff University, Cardiff, United Kingdom

**Keywords:** FM1-43, mechano-electrical transducer channel, hair cell, zebrafish, hearing, ototoxicity

## Abstract

The styryl dye FM1-43 is widely used to study endocytosis but behaves as a permeant blocker of the mechano-electrical transducer (MET) channel in sensory hair cells, loading rapidly and specifically into the cytoplasm of hair cells in a MET channel-dependent manner. Patch clamp recordings of mouse outer hair cells (OHCs) were used to determine how a series of structural modifications of FM1-43 affect MET channel block. Fluorescence microscopy was used to assess how the modifications influence hair-cell loading in mouse cochlear cultures and zebrafish neuromasts. Cochlear cultures were also used to evaluate otoprotective potential of the modified FM1-43 derivatives. Structure-activity relationships reveal that the lipophilic tail and the cationic head group of FM1-43 are both required for MET channel block in mouse cochlear OHCs; neither moiety alone is sufficient. The extent of MET channel block is augmented by increasing the lipophilicity/bulkiness of the tail, by reducing the number of positive charges in the head group from two to one, or by increasing the distance between the two charged head groups. Loading assays with zebrafish neuromasts and mouse cochlear cultures are broadly in accordance with these observations but reveal a loss of hair-cell specific labelling with increasing lipophilicity. Although FM1-43 and many of its derivatives are generally cytotoxic when tested on cochlear cultures in the presence of an equimolar concentration of the ototoxic antibiotic gentamicin (5 µM), at a 10-fold lower concentration (0.5 µM), two of the derivatives protect OHCs from cell death caused by 48 h-exposure to 5 µM gentamicin.

## Introduction

**FIGURE 1:**
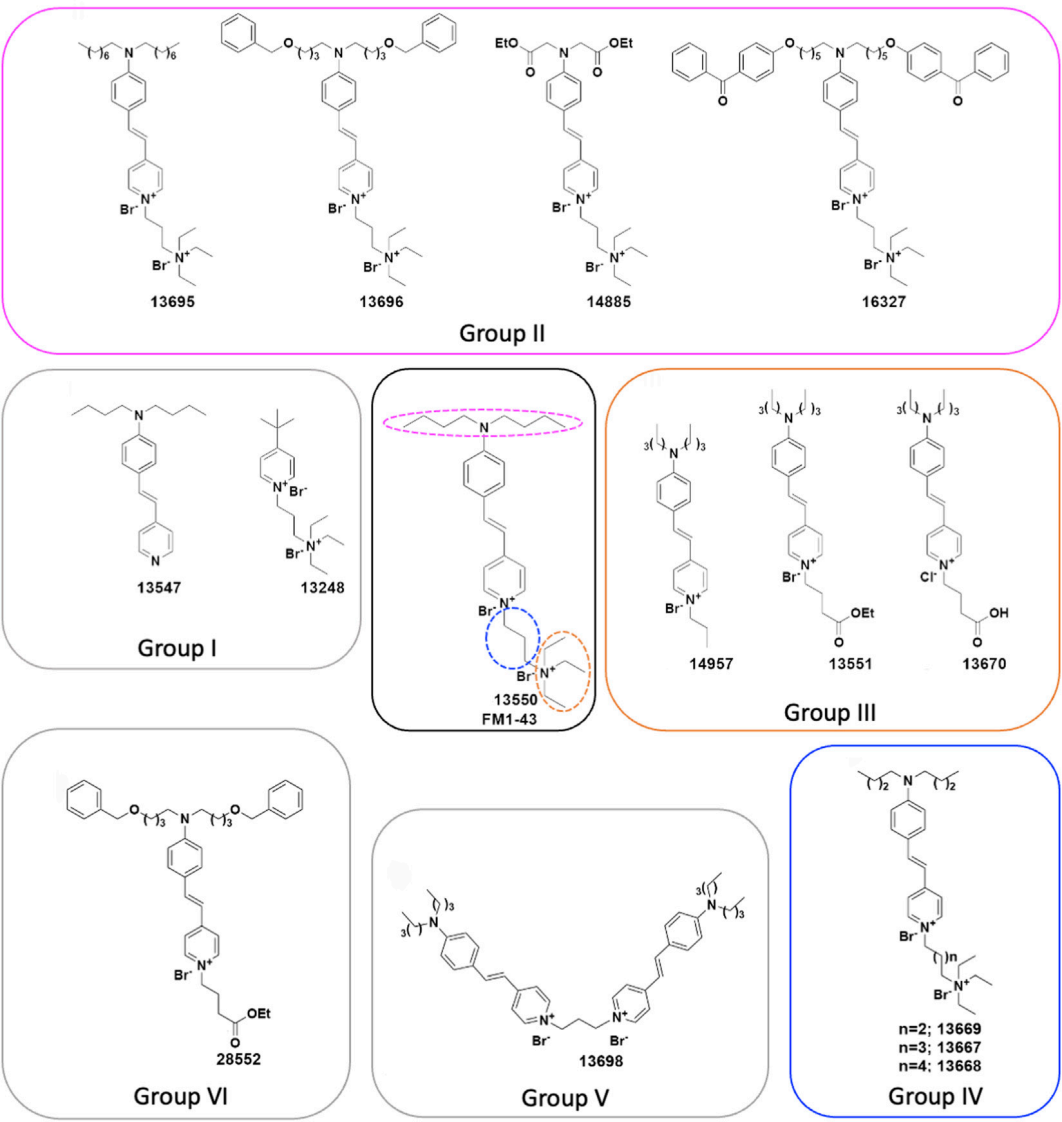
Chemical structures of FM1-43 (13550) and its derivatives. (Centre) Structure of FM1-43 (13550). Coloured dashed lines indicate the three regions modified in the Group II (magenta), Group III (orange) and Group IV (blue) derivatives. (Centre-left) Group I compounds with truncations of either the head or tail region, (Top) Group II compounds with lipophilic tail modifications, (Centre-right) Group III compounds with hydrophilic head modifications, and (Bottom-right) Group IV compounds with different length linkers between the quaternary nitrogens. (Bottom) Group V compound 13698 is a bis-derivative, and (Bottom-left) the Group VI compound 28852 combines modifications to both the lipophilic tail and the hydrophilic head.

FM dyes are a valuable class of water-soluble fluorescence probes. The chemical structure of FM1-43 consists of three distinct moieties (see Figure 1 centre): a lipophilic tail, exemplified by two linear alkyl chains (magenta); a central core consisting of a pyridine ring conjugated via at least one double-bond linker to a phenyl ring; and a hydrophilic head (orange) containing a quaternary nitrogen, linked to the pyridinium nitrogen via an alkyl chain (blue). These moieties play different roles and contribute differently to the behaviour of the dyes in living cells: the hydrophobic tail facilitates the partitioning into membranes; the conjugated aromatic systems underlie the broad fluorescence spectral properties of these dyes; and the hydrophilic cationic head prevents the permeation through the membrane (Betz et al., 1996). FM dyes were first described (and are still widely used) as tools for following endo- and exo-cytosis, particularly at synapses in living systems, as well as for studying the trafficking of endosomes (Betz et al., 1996; Betz et al., 1992; Betz and Bewick, 1992). The distinctive property of this class of dyes is that they partially permeate into the outer layer of the membrane (Betz et al., 1992). The partitioning of the compound into the phospholipidic layer results in an increased fluorescence and has allowed endocytosis, the main mechanism whereby the compound is internalised in many cells, to be followed and studied (Betz et al., 1996).

It has been reported, however, that these dyes are able to enter via other mechanisms into certain types of cells. A considerable body of evidence indicates that FM1-43 is selectively able to enter into the cytoplasm of mechanosensory hair cells, whilst only labelling the plasma membrane of surrounding non-sensory supporting cells, in different systems such as the lateral line organs of *Xenopus* (Nishikawa and Sasaki, 1996) and zebrafish larvae (Seiler and Nicolson, 1999), the bullfrog sacculus (Gale et al., 2000), and the mouse cochlea (Gale et al., 2001). These studies indicate that the mechanism of entry of FM1-43 into sensory hair cells is via the mechano-electrical transducer (MET) channel (Nishikawa and Sasaki, 1996; Gale et al., 2001; Meyers et al., 2003), a non-selective, mechanically-gated cation channel located at the tips of the stereocilia (Kros et al., 1992; Kawashima et al., 2011; Fettiplace and Kim, 2014). In addition, it has also been reported that FM1-43 enters cells expressing TRPV1 or P2X2 channels in the presence of their agonists (Meyers et al., 2003). FM4-64, another member of this dye family, was found to enter astrocytes via store-operated calcium channels and TRP channels, with the results indicating the main entry into these cells is via a non-endocytic mechanism (Li et al., 2009). Interestingly, the MET channel is also the main mechanism of entry of aminoglycoside antibiotics into sensory hair cells (Gale et al., 2001; Marcotti et al., 2005; Wang and Steyger, 2009; Alharazneh et al., 2011). Once the aminoglycosides have accumulated into these cells, they trigger a cascade of cellular responses that lead to hair-cell death and irreversible hearing loss, an unfortunate and unwanted side effect that is reported in ∼25% of all patients treated with these medications (Rizzi and Hirose, 2007; Huth et al., 2011).

In this study, we investigate how variation in the three moieties of FM1-43 and modulation of the dye’s physicochemical properties influence its interaction with the MET channel, and its selective loading into the hair cells of both zebrafish lateral line organs and mouse cochlear cultures. Furthermore, as MET channel block by a number of compounds is known to offer protection against aminoglycoside damage (Ou et al., 2010; Kenyon et al., 2017; Kirkwood et al., 2017; Chowdhury et al., 2018; Kitcher et al., 2019; O’Reilly et al., 2019; Kenyon et al., 2021), we investigate if any of the FM1-43 derivatives are also able to offer any protection against gentamicin damage in mouse cochlear cultures.

## Materials and methods

### Chemistry

All commercial reagents were of the highest available purity and purchased from Sigma-Aldrich, Alfa Aesar, Apollo Scientific, Fluorochem or Tokyo Chemical Industry. Unless otherwise stated, chemicals were used as supplied without further purification. Anhydrous solvents were purchased from Acros (AcroSeal™) or Sigma-Aldrich (SureSeal™) and stored under nitrogen. Petroleum ether refers to the fraction with a boiling point between 40°C and 60°C. Thin Layer Chromatography (TLC): pre-coated aluminium-backed plates (60 F254, 0.2 mm thickness, Merck) were visualized under both short- and long-wavelength UV light (254 and 366 nm). Flash column chromatography was carried out using commercial pre-packed columns from Biotage, Isco, Grace or columns filled with Merck silica gel 60 (40–63 µm), or columns packed with C18 silica on an ISCO Combiflash Rf or a Biotage Isolera Prime. High performance liquid chromatography (HPLC) purification was performed on an Agilent 1,100 series HPLC spectrometer, using a Phenomenex Luna 10 μm C18 150 mm × 15 mm column. Compounds were eluted using water and acetonitrile at 15 mL/min, and detected at 254 nm.

Proton nuclear magnetic resonance (NMR) spectra were recorded at 500 MHz on a Varian VNMRS 500 MHz spectrometer or at 600 Mz on a Varian VNMRS 600 MHz spectrometer at 30°C, using residual isotopic solvent (CHCl_3_, *δ* = 7.27 ppm, DMSO *δ* = 2.50 ppm) as an internal reference. Chemical shifts are quoted in parts per million (ppm). Coupling constants (J) are recorded in Hertz (Hz). The following abbreviations are used in the assignment of NMR signals: s (singlet), d (doublet), t (triplet), q (quartet), qn (quintet), m (multiplet), bs (broad singlet), dd (doublet of doublet), dt (doublet of triplet). Carbon NMR spectra were recorded at 125 and 151 MHz on Varian 500 and 600 MHz spectrometers respectively and are proton decoupled, with residual isotopic solvent (CHCl_3_, *δ* = 77.00 ppm, DMSO *δ* = 39.52 ppm) as an internal reference.

Liquid chromatography–mass spectrometry (LCMS) data was recorded on a Waters 2695 HPLC using a Waters 2487 UV detector and a Thermo LCQ ESI-MS. Samples were eluted through a Phenomenex Lunar 3 μm C18 50 mm × 4.6 mm column, using water and acetonitrile acidified by 0.1% formic acid at 1 mL/min and detected at 254 nm. The following methods were used: method 1: water (+0.1% formic acid)/acetonitrile (+0.1% formic acid) = from 65/35 to 10/90 in 3.5 min, then isocratic 10/90 0.4 min, then from 10/90 to 65/35 in 0.1 min; method 2: water (+0.1% formic acid)/acetonitrile (+0.1% formic acid) = from 70/30 to 10/90 in 5 min, then isocratic 10/90 1.0 min, then from 10/90 to 70/30 in 0.5 min and then isocratic 70/30 for 0.5 min.

LCMS (Mass Directed Auto Purification): LCMS data was recorded on a Shimatzu Prominence Series coupled to a LCMS-2020 ESI and APCI mass spectrometer. Samples were eluted through a Phenomenex Gemini 5 μm C18 110A 250 mm × 4.6 mm column, using water and acetonitrile acidified by 0.1% formic acid at 1 mL/min and detected at 254 nm. The following method, marked as method 3, was used: water (+0.1% formic acid)/acetonitrile (+0.1% formic acid) = isocratic 95/5 1 min, then from 95/5 to 5/95 in 20 min, then isocratic 5/95 for 4 min, then from 5/95 to 70/30 in 5 min.

Physicochemical properties were calculated using MarvinSketch 16.8.15.0 by ChemAxon (https://www.chemaxon.com). Compound purity was assured by a combination of high field multinuclear NMR (H, C) and HPLC; purity by the latter was always >95%.

Synthetic schemes and procedures are reported in the Supplementary Material (Supplemental Chemical Synthesis).

### Biology

#### Zebrafish hair cell loading assay

Zebrafish embryos were obtained from sibling crosses of adult nacre (*mifta−/−*) fish (Lister et al., 1999) housed at the University of Sussex. The nacre strain has substantially reduced numbers of pigmented melanophores and fluorescent signal detection is thus improved. The development of lateral line neuromasts and hair cells in this strain appears normal (Kenyon et al., 2017). Larvae at 4 days post fertilisation (dpf) were dispensed into 96-well microtiter plates at a density of 3–4 per well in 50 µL of E3 medium (1 mM NaCl, 0.17 mM KCl, 0.33 mM MgSO_4_, 0.33 mM CaCl_2_). Fish were exposed to FM1-43 (13550) and derivatives at concentrations of 10, 3, 1, and 0.3 µM for times of 1, 2.5, 5, and 10 min. Larvae were washed three times in E3, anaesthetised with 0.025% MS222 (MilliporeSigma E10521) and imaged immediately. Images were taken of the fourth neuromast of the posterior lateral line (P4) using a Nikon D5000 camera attached to a Zeiss IM-35 inverted microscope. All images were taken at the same exposure. At least three independent experiments were performed for each derivative. To ensure consistency in each experiment the parent compound, 13550, was imaged each time an experiment was run, therefore for 13550 *n* = 8. For full details of analysis, see Supplementary Material: Methods, Results and Figures.

#### Mouse cochlear culture preparation

Tissues obtained from wildtype CD-1 mice (Charles Rivers, United Kingdom) of either sex, at postnatal day 2 (P2), were used for the preparation of the mouse cochlear cultures. Cochlear cultures were prepared as previously described (Russell and Richardson, 1987). In brief, P2 pups were killed by cervical dislocation and surface sterilized by three 1 min washes in 80% ethanol. Subsequent dissections were performed in Hanks’ balanced salt solution (HBSS; Thermo Shandon 14025050) buffered with 10 mM HEPES pH 7.0 (Sigma H0887) (HBHBSS). Cochleae were removed from the labyrinth and explanted onto collagen-coated (Corning 354236) coverslips and immersed in rat cochlear culture media (RCM; 93% DMEM-F12, 7% foetal bovine serum and 10 μg mL^–1^ ampicillin), sealed in Maximow slide assemblies, and left to adhere to the collagen for 24 h at 37°C.

#### Mouse cochlear culture protection assay

Following 24 h incubation, coverslips with adherent cochleae were removed from the Maximow slide assemblies, placed in 35 mm Petri dishes (Greiner Bio-One 627161) and incubated for 48 h at 37°C in a 5% CO_2_ incubator in the presence of 1 mL RCM/DMEM-F12 (1:4) containing either vehicle (0.5% DMSO), 5 μM gentamicin (Sigma G3632) and 0.5% DMSO, or 5 μM gentamicin along with selected concentrations of either FM1-43 or its derivatives (dissolved in DMSO). Following 48 h incubation, cultures were washed twice in phosphate-buffered saline, fixed in 3.7% formaldehyde (Sigma F1635), and stained with TRITC-phalloidin (Sigma P1951). Cultures were mounted on glass slides in Vectashield (Vector Laboratories H-1000) and imaged on a Zeiss Axioplan microscope with a 40× objective (0.75 NA) using a Spot RT slider digital camera. Compounds that provided protection were each re-tested on 3–7 individual occasions. Numbers of outer hair cells (OHCs) were counted in a 221 μm (1,200 pixel) length segment of the organ of Corti. For full details of analysis, see Statistics section below.

#### Electrophysiology on mouse cochlear cultures

MET currents were recorded and analyzed using previously described methods (Kirkwood et al., 2017). In brief, OHCs in organotypic cultures prepared from P2 CD-1 mice were studied, with recordings performed in cultures that had been maintained for 1–2 days *in vitro*. The cultures were placed in a microscope chamber that was continuously perfused with extracellular solution containing (in mM): 135 NaCl, 5.8 KCl, 1.3 CaCl_2_, 0.9 MgCl_2_, 0.7 NaH_2_PO_4_, 5.6 D-glucose, 10 HEPES-NaOH, 2 sodium pyruvate. MEM amino acids solution (50X) and MEM vitamins solution (100X) were added to a final concentration of 1X from concentrates (Fisher Scientific). The pH was adjusted to 7.48 with 1 M NaOH (osmolality ∼308 mOsmol kg^-1^). MET currents were recorded using the whole-cell configuration of the patch clamp technique both before and during exposure to different compounds at membrane potentials ranging from −164 to +96 mV. The compounds were locally superfused onto the OHC recorded from in a solution containing (in mM): 145 NaCl, 5.8 KCl, 1.3 CaCl_2_, 0.9 MgCl_2_, 0.7 NaH_2_PO_4_, 5.6 glucose, 10 HEPES-NaOH, 2 sodium pyruvate. The pH was adjusted to 7.48 with NaOH (osmolality ∼305 mOsmol kg^-1^). This solution, without compound, was superfused as a control solution before and after the application of each compound. The intracellular solution was composed of (in mM): 137 CsCl, 2.5 MgCl_2_, 1 EGTA-CsOH, 2.5 Na_2_ATP, 10 sodium phosphocreatine, 5 HEPES-CsOH; pH adjusted to 7.3 with CsOH (osmolality ∼295 mOsmol kg^-1^). Currents were elicited by stimulating the OHC hair bundles using a fluid jet from a pipette (tip diameter 8–10 μm) driven by a piezoelectric disc (Kros et al., 1992; Marcotti et al., 2005). Mechanical stimuli (filtered at 1.0 kHz, 8-pole Bessel) were applied as 45 Hz sinusoids with driver voltage amplitudes of ±40 V. Currents were acquired using pClamp (Molecular Devices) or Signal 4.3 (Cambridge Electronic Design) software and stored on computer for off-line analysis. For all recordings, series resistance compensation was applied (60%–80%), and, as the maximum voltage drop across the residual series resistance was estimated to be less than 5 mV, voltage values were not corrected for this small error. A correction of −4 mV was applied to reported voltages to account for the liquid junction potential between extra- and intracellular solutions.

Dose-response curves were fitted with the equation:

$$\frac{I}{I_{C}}=\frac{1}{1+{\left(\left[B\right]/K_{D}\right)}^{n_{H}}}$$ (1)

where *I*_*C*_ is the control current in the absence of the compound, [*B*] is the concentration of the blocking compound, *K*_*D*_ is the half-blocking concentration, and *n*_*H*_ is the Hill coefficient. Means are quoted ±SEM.

#### Dye loading assay in mouse cochlear culture hair cells

Coverslips with adherent cultures were removed from the Maximow slide assemblies, placed in a Perspex viewing chamber and were treated with 0.3 μM of either FM1-43 (13550) or one of the FM1-43 derivatives in 500 μL HBHBSS. Images were captured using a x60 dipping lens at a fixed distance from the basal end of the explant at 5, 10, and 15 min after the application of compound. Intensity values were obtained from 40 × 40-pixel regions of interest in 10 adjacent OHCs and means calculated for statistical analysis. Each compound was tested on 3 to 9 occasions. For details of analysis, see Supplementary Material: Methods, Results and Figures.

#### Data analysis for mouse cochlear culture protection assay

Cell counts were modelled as a generalised Poisson process, using generalised linear mixed models. Separate models were applied to 0.5 and 5 μM treatments. Treatments were fitted as categorical fixed effects, nested within experiment dates (date as a blocking factor modelled as a random effect). Compounds showed different levels of variation, which was included in the model, also nested within date blocks but assumed not to vary between dates. Pairwise contrasts were assessed between each drug and both the control and the gentamicin only treatment. Statistical analyses were conducted using R, version 4.1.2 (R Core Team, 2019). Additional R packages were: glmmTMB (Brooks et al., 2017) for Mixed effects model, AICcmodavg (Mazerolle, 2020) for model selection, and ggplot2 (Wickham, 2016) for data visualisation.

## Results

### Chemical modifications of FM1-43

The FM1-43 derivatives considered in this study were prepared according to the synthetic procedures reported in the Supplementary Material. FM1-43 (Figure 1 centre) was also synthesized in house in order that the production process was similar to that used to prepare the derivatives and is henceforth referred to as 13550. To understand which moieties of FM1-43 are required for the interaction with the MET channel, and how modifications affect the physicochemical properties of the dye, six classes of derivative were synthesised. These are referred to as Groups I-VI, and their structures are shown in Figure 1.

The two compounds in Group I (Figure 1 centre-left) were designed to test the properties of derivatives bearing only the lipophilic (13547) or the hydrophilic moiety (13248). The four compounds in Group II (Figure 1 top) were produced to investigate how variations in lipophilicity and/or the insertion of bulky substituents in the alkyl chain would affect activity. The design of these compounds was driven by results previously reported for FM3-25, an FM1-43 analogue which bears a very long lipophilic chain, does not block the MET current (Gale et al., 2001; van Netten and Kros, 2007), and is not able to permeate into hair cells (Meyers et al., 2003). Compound 13695 has a longer alkyl tail chain compared to FM1-43 which results in an increased lipophilicity. Compounds 13696 and 16327 have bulky substituents and an increased lipophilicity with 16327 carrying two benzophenones, chemical features that are commonly used for photoaffinity labelling. Compound 14885, with two ester moieties, has instead a reduced lipophilicity and carries the esterified function present in the calcium chelator BAPTA.

The role of the hydrophilic tetraethylammonium moiety, the quaternary nitrogen carrying one of the two positive charges, was investigated with the three Group III compounds (Figure 1 centre-right) in which it was substituted by an alkyl chain (14957), an alkyl ester moiety (13551), and an alkyl acid (13670). Compounds 14957 and 13551 only have one positive charge and increased lipophilicity, whereas the alkyl acid in 13670 is negatively charged at physiological pH making a zwitterionic molecule (one positive charge at the nitrogen and one negative charge at the carboxylic acid moiety). Subsequently, three Group IV compounds (Figure 1 bottom-right) were generated to investigate the role of the spacer between the two positive charges by increasing its length from three carbons (as in the parent compound) to four (13669), five (13667), and six (13668) carbons. The final two modifications reported in Figure 1 were designed following results from experiments on the other derivatives. These are Group V compound 13698, a symmetrical bis-FM1-43 derivative (Figure 1 bottom), and Group VI compound 28552 (Figure 1 bottom-left). The rationale behind the design of compound 13698 was driven by the fact that FM1-43 has a Hill coefficient of 2, suggesting that two molecules of FM1-43 are needed to block the MET channel as described previously (Gale et al., 2001); hence we linked two molecules of FM1-43 in one structure (Figure 1 bottom). Compound 28552 (Figure 1 bottom-left) was designed based on results obtained for compound 13696 (Group II) and compound 13551 (Group III) and it carries modifications at both the lipophilic and hydrophilic moieties.

### MET channel electrophysiological profile of FM1-43 and its derivatives

**FIGURE 2:**
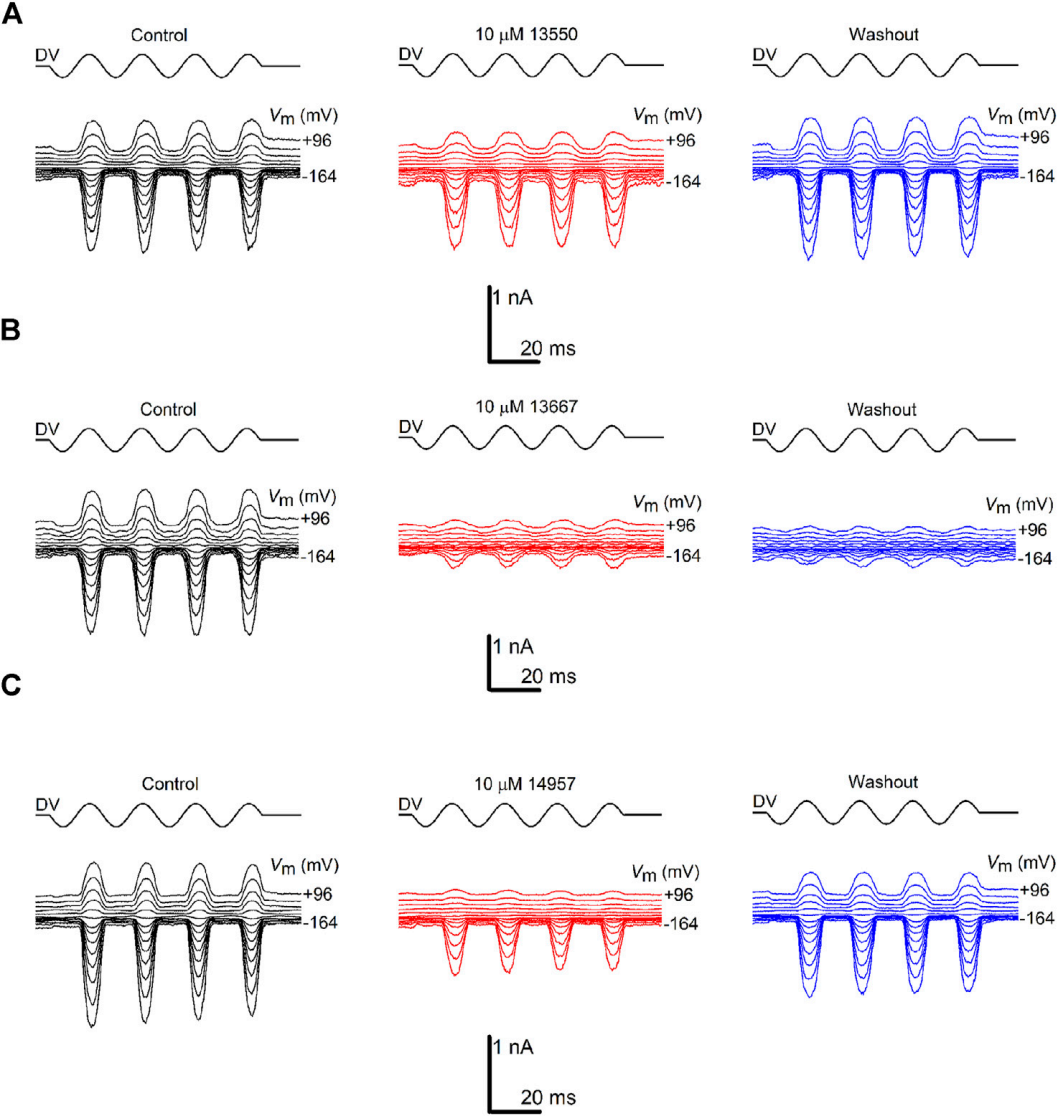
Varying degrees of MET channel block by FM1-43 and its derivatives. MET currents recorded from OHCs, before (black traces), during (red traces) and after (blue traces) extracellular exposure to **(A)** 10 µM FM1-43 (13550) **(B)** 10 µM 13667 **(C)** 10 µM 14957. The currents were recorded at membrane potentials ranging from −164 to +96 mV. MET channel opening and closing was driven by a sinusoidal stimulus delivered to the fluid-jet (shown above each trace; driver voltage, DV). Currents are reduced during exposure to FM1-43 (13550) and the derivatives, both at hyperpolarized and depolarized potentials, with the degree of block varying between the compounds. Recovery of the currents during washout was observed for 13550 **(A)** and partial recovery for 14957 **(C)**, but not for 13667 **(B)**. Experiments were performed at room temperature (20°C–22°C).

**FIGURE 3:**
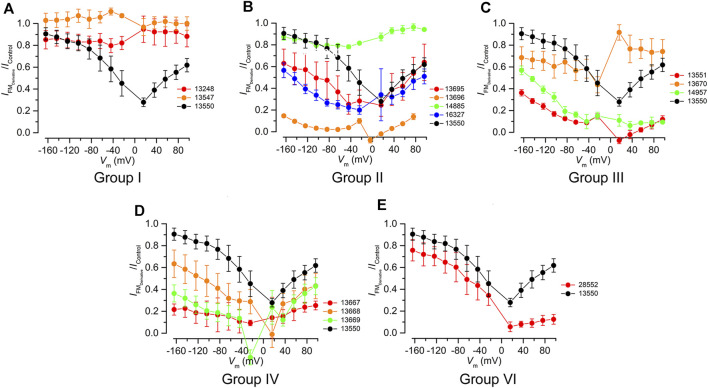
Fractional block of the MET currents by FM1-43 and its derivatives grouped in chemical classes using FM1-43 (13550) as a reference (black). **(A)** Truncated Group I compounds, **(B)** Group II compounds with modified lipophilic tails **(C)** Group III compounds with hydrophilic head modifications, **(D)** Group IV with varying length linkers of the quaternary nitrogens, and **(E)** Group VI compound with modifications to both the lipophilic tail and the hydrophilic head. All compounds were tested at 10 μM, except compound 13696, which was tested at 3 µM.

To investigate how the different chemical modifications affect interactions with the MET channel, all of the derivatives (with the exception of 13698 which precipitated during bath application) were tested for their ability to block MET currents and compared with the block obtained with 13550 (the in-house synthesised FM1-43). Examples of MET currents observed with 13550, Group IV compound 13667, and Group III compound 14957 before, during and after application are shown in Figures 2A–C. The fractional block of the MET currents organised by structural modification (Groups I-VI), is shown in Figures 3A–E with data from the parent compound 13550 included in each panel for comparison. Each compound was tested at membrane potentials ranging from −164 to +96 mV at a concentration of 10 μM, except for the strongest blocker, 13696, which was only tested at 3 μM. As reported in previous studies (Gale et al., 2001) and as confirmed in the current study, FM1-43 acts as a permeant blocker of the MET channel showing the strongest block at +16 mV, a block that is released at extreme positive and negative membrane potentials.

The two compounds from Group I, 13547 and 13248, in which either the charged head or the alkyl tail of parent compound 13550 has been truncated (Figure 1 centre-left), did not block the MET current at any potential tested (Figure 3A). All Group II compounds (Figure 1 top) retain two positive charges in their structure, which should facilitate their interaction with the channel, but the lipophilic moiety is modified and may therefore modulate the permeability via the channel. The effects of these compounds on the level of MET channel block as a function of membrane potential are shown in Figure 3B. Compound 13695, with longer chains compared to the parent compound and an increased lipophilicity, showed a similar profile to that of 13550 at positive membrane potentials with the strongest block observed at +16 mV. At negative membrane potentials however 13695 showed a stronger block of the MET current than 13550, i.e., there was a less pronounced release of the block with increasing hyperpolarization. A similar block was obtained with 16327, a compound that has a greatly increased lipophilicity and bulky substituents at the end of the chain. The finding that there was still a release of the block at negative membrane potentials shows that 13695 and 16327 are permeant blockers, but suggests that their larger lipophilic tails slow entry into the cells under the electrical driving force compared to the parent compound. Group II derivative 13696 (Figure 1 top) carries a bulky moiety (two additional phenyl rings) but, due to the presence of the two oxygens, has a less pronounced increase in lipophilicity compared to the two previously described compounds. This compound showed the strongest block of the MET channel and exhibited a strong block of the MET current even at 3 μM, when compared with 13550 at 10 μM (Figure 3B). Derivative 14885, the only compound from the Group II series that is more hydrophilic than the parent compound, did not block the MET current at any potential tested (Figure 3B).

Group III compounds (Figure 1 centre-right) were used to investigate how removal of the terminal positively charged nitrogen affects modulation of the MET current. Compounds with three different substituents were tested: an alkyl chain (14957), an ester (13551) and a carboxylic acid (13670) with the first two having one positive charge, and the latter being, overall, a neutral molecule, acting as zwitterion with one positive and one negative charge. Modification of this terminal moiety gave unexpected results; both 14957 and 13551 showed a stronger block of the MET current compared to 13550 with a voltage-dependent pattern and a progressive release of the block at increasingly negative membrane potentials indicating that both compounds still are permeant blockers of the MET channel (Figure 3C). Furthermore, 13670, carrying both negatively (carboxylic) and positively (pyridinium) charged moieties was still able to provide a block of the MET current, albeit with a very different voltage-dependent pattern compared to that of the parent compound. It was not able to block the channel at positive potentials but started to block the MET current at negative potentials, with the strongest block at approximately −20 mV. This was followed by a slight release of the block with further hyperpolarization, which is still greater than the block provided by FM1-43.

Compounds from Group IV were used to look at the role of the spacer between the two positive charges: this consists of three carbons in FM1-43 and was increased to four (in 13669), five (in 13667) and six (in 13668) (Figure 1 bottom-right). These compounds are still able to interact with the MET channel (Figure 3D) and offered a more pronounced block of the current compared to 13550. The most effective compound was 13667 that, with a five-carbon spacer, gave ∼80% block across a wide range of membrane potentials. The least effective compound of this series was 13668 which has a six-carbon spacer between the positive charges.

The Group V bis-FM1-43 derivative 13698 (Figure 1 bottom) could not be tested using electrophysiology techniques due to the compound crystallising on the cochlear cultures, preventing patch clamp seal formation.

Group VI compound 28552 carries the same lipophilic tail as 13696 and, like 13551, only has one positive charge (Figure 1 bottom-left). It was tested to see if these modifications would further modulate the interaction with the channel and provide a non-permeant block. The compound, however, did not have the desired profile and was found to be only slightly more effective compared to 13550 (Figure 3E) whilst still acting as a permeant blocker of the MET channel.

**FIGURE 4:**
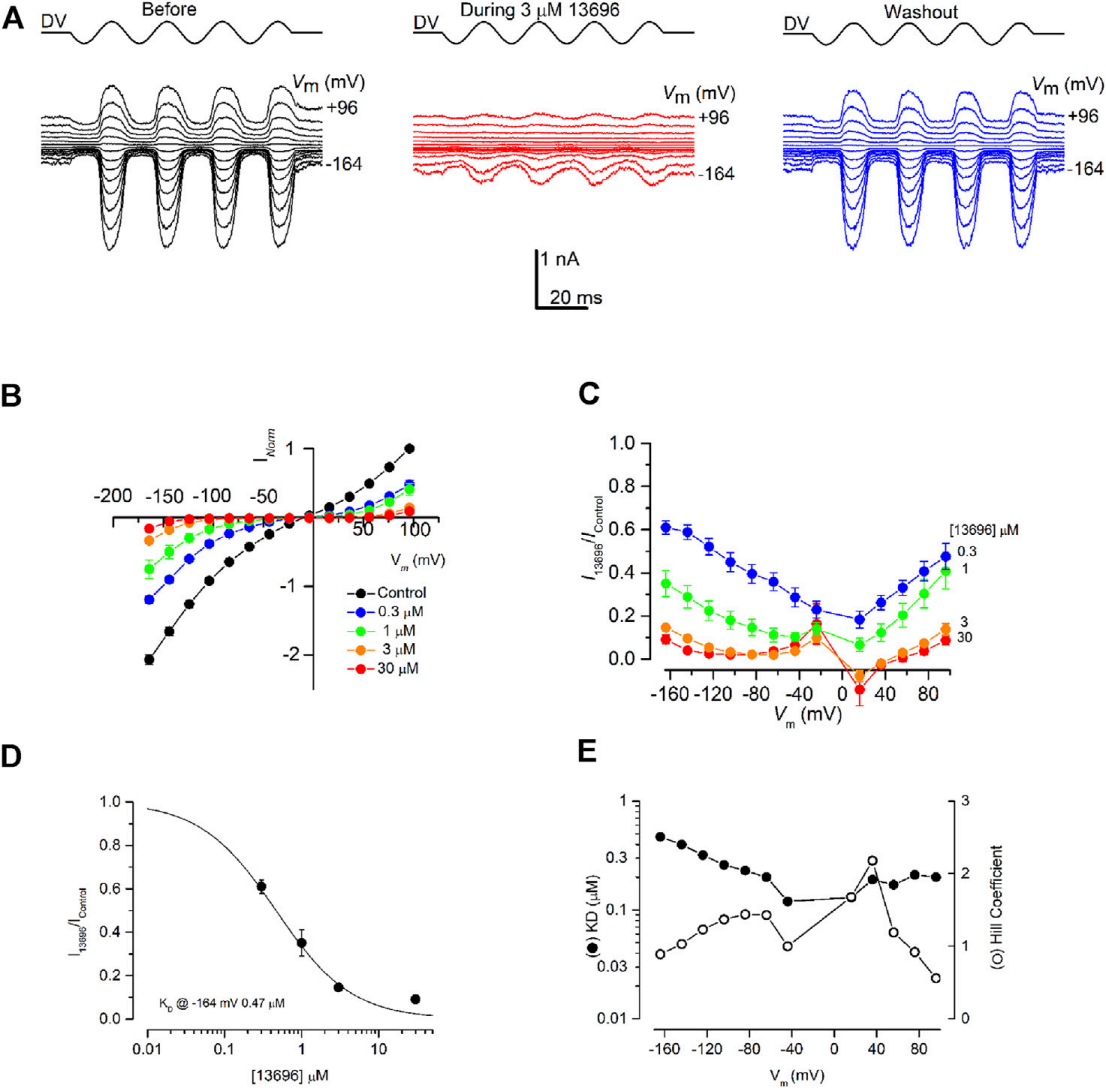
MET channel block by FM1-43 derivative, 13696. **(A)** MET currents recorded from an OHC before (black trace), during (red trace) and after (blue trace) extracellular exposure to 3 µM 13696. Membrane potentials were stepped between −164 and +96 mV and MET currents simultaneously recorded in response to a sinusoidal stimulus delivered to the fluid-jet (driver voltage, DV) shown above each trace. 13696 reduced MET currents at both hyperpolarized and depolarized potentials with the block fully reversible as seen from the washout. **(B)** Average normalized current-voltage curves for varying concentrations of 13696 (0.3–30 µM). Currents were normalized to the peak control current measured at +96 mV. The block increases at both depolarized and hyperpolarized potentials with increasing concentration of the compound. **(C)** Average fractional block curves showing the current during 13696 exposure (0.3–30 µM) relative to the control current at each membrane potential. For all concentrations of 13696, the block is strongest at the intermediate membrane potentials with some release of the block at the extreme depolarized and hyperpolarized potentials. **(D)** Dose-response function from the currents measured at −164 mV, revealing a half-blocking concentration of 0.47 μM at this membrane potential. **(E)** Equilibrium dissociation constants (K_D_) and Hill coefficients obtained from dose-response functions derived from the currents measured at each membrane potential. K_D_ values vary between 0.12 and 0.47 µM and the Hill coefficient ranges from 0.56 to 2.18. Numbers of cells **(B)** Control, 20; 0.3 µM, 6; 1 μM, 6; 3 μM, 4; 30 μM, 4 **(C)** 0.3 µM, 6; 1 μM, 6; 3 μM, 4; 30 μM, 4 **(D)** Between 4 and 6 cells were used for each data set. Means are shown ±SEM.

The most promising derivative 13696 (Figure 1 top, Group II) was tested further at concentrations ranging from 0.3 to 30 μM (Figure 4). Compound 13696 showed a strong, fully reversible, block at 3 μM (Figure 4A), and blocked the MET currents in a concentration- and voltage-dependent manner, as can be seen in the current-voltage (Figure 4B) and the fractional block (Figure 4C) plots. Like the parent compound FM1-43 (Gale et al., 2001), block of the MET current was strongest at +16 mV, with release of the block not only at depolarized but also at hyperpolarized potentials indicating that this compound is also a permeant blocker of the channel (Figure 4C). An example of a dose-response curve (at −164 mV, close to the electrical driving force across the MET channel in physiological conditions) is plotted in Figure 4D, revealing a half-blocking concentration (K_D_) of 0.47 μM and a Hill coefficient of 0.89. The K_D_ and the Hill coefficient (Figure 4E) vary with voltage in a similar manner as previously reported for FM1-43, but with block an order of magnitude stronger (Gale et al., 2001).

### Loading of FM1-43 and its derivatives in zebrafish lateral line neuromasts and mouse cochlear cultures

**FIGURE 5:**
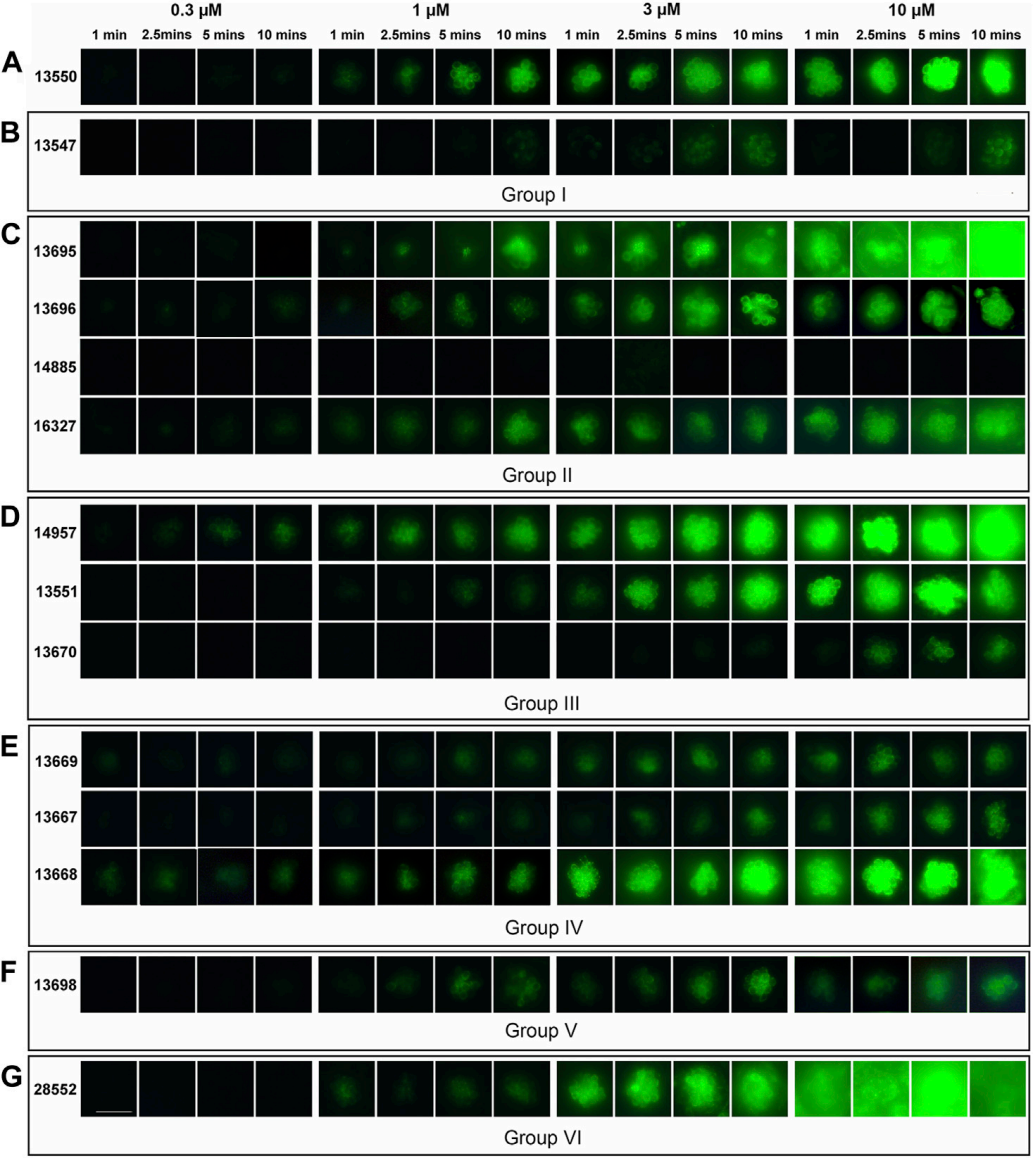
Loading of FM1-43 and its derivatives into zebrafish lateral line hair cells. Four-day post fertilisation zebrafish larvae were exposed to FM1-43 and its derivatives at concentrations of 0.3, 1, 3 and 10 μM for either 1, 2.5, 5 or 10 min before washout and imaging. Images are representative of larvae treated with **(A)** 13550 - parent compound, **(B)** truncated Group I structures **(C)** Group II compounds with lipophilic tail modifications, **(D)** Group III compounds with hydrophilic head modifications, **(E)** Group IV with varying length linkers of the quaternary nitrogens, **(F)** the bis-derivative Group V compound and **(G)** the Group VI compound with modifications to both the lipophilic tail and the hydrophilic head. Images are of P4 in the posterior lateral line. Scale bar = 25 µm.

The ability of the FM1-43 derivatives to load into the hair cells of the zebrafish lateral line was tested using, for each compound, four different concentrations (0.3, 1, 3, and 10 µM) at four different time points (1, 2.5, 5, and 10 min). The parent compound 13550 (Figure 5A) and thirteen of the fourteen FM1-43 derivatives from Groups I-VI (Figures 5B–G) were compared. Compound 13248 was excluded as it is not fluorescent and cannot be detected in this assay. Representative images of neuromast loading at the different concentrations and time points are shown in Figure 5.

Compound 13550, like commercially available FM1-43, shows a strong time- and concentration-dependent fluorescence in the cytoplasm of the neuromast hair cells and no label in the nucleus (Figure 5A). In contrast, Group I compound 13547, which has only the lipophilic moiety, showed little loading except at highest concentrations and longest time points (Figure 5B). Of the compounds in the other groups, Group III compound 14957 (Figure 5D) and Group IV compound 13668 (Figure 5E) both loaded at the lowest concentration tested, Group II compound 14885 with the hydrophilic tail failed to load at any concentration (Figure 5C), and a total of four compounds from different groups loaded considerably less well at the highest concentration and/or across most of the concentration range; these included Group III compound 13670, Group IV compounds 13669 and 13667, and Group V compound 13698 (Figures 5D–F, respectively). With five of the compounds, a loss of specificity for hair-cell labelling was observed with supporting cells within the neuromasts and surrounding epidermal cells also loading with the dye. These derivatives were Group II compounds 13695 and 16327, Group III compound 14957, Group IV compound 13668, and Group VI compound 28552 (Figures 5C–F respectively).

**FIGURE 6:**
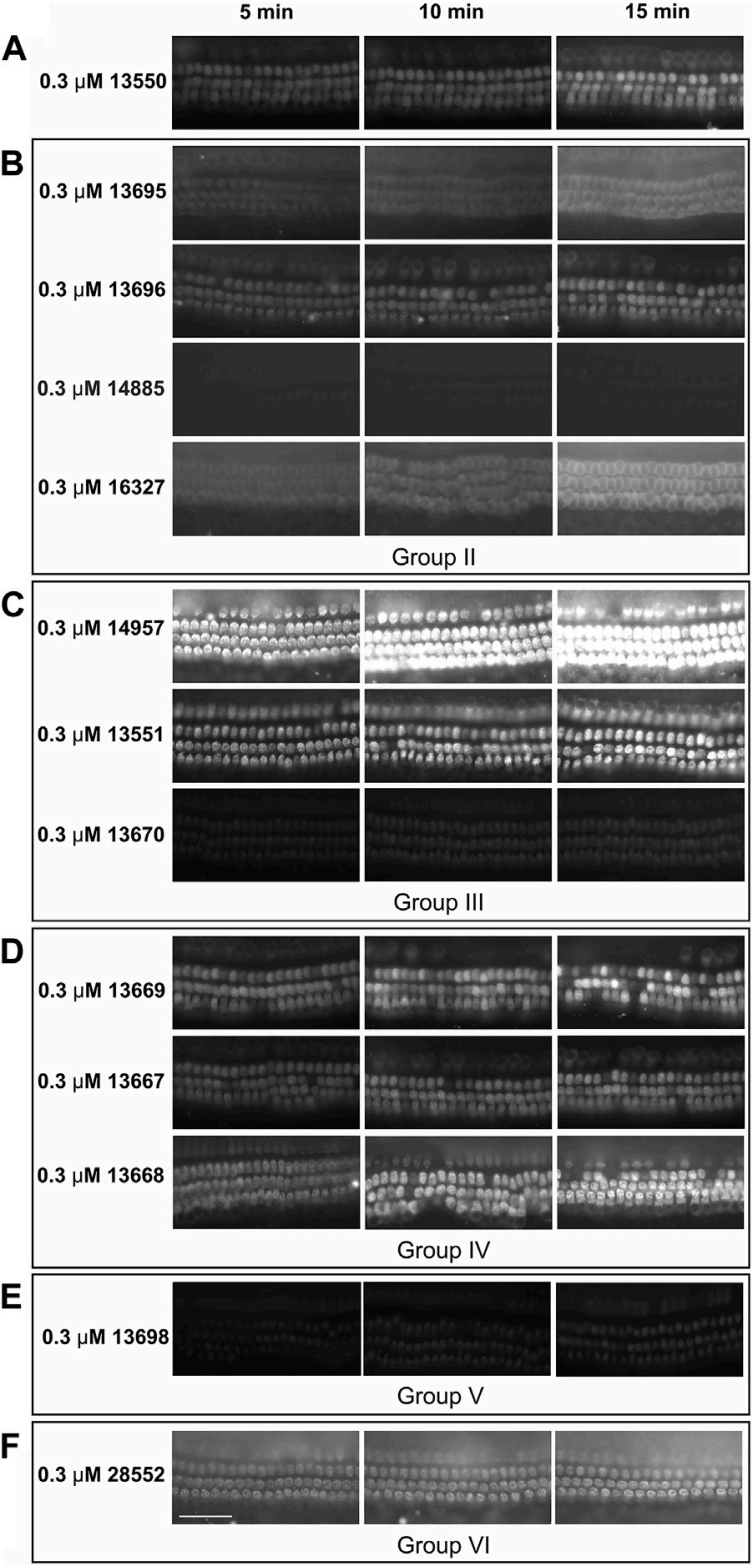
Loading of FM1-43 and its derivatives into mouse cochlear cultures. Postnatal day 2 mouse cochlear cultures were exposed to FM1-43 and its derivatives at a concentration of 0.3 μM and imaged at 5, 10, and 15 min after their addition. Images are representative of cultures treated with: **(A)** 13550 - parent compound **(B)** Group II compounds with lipophilic tail modifications, **(C)** Group III compounds with hydrophilic head modifications **(D)** Group IV compounds with linkers of varying length between the quaternary nitrogens, **(E)** the Group V bis-derivative and **(F)** the Group VI compound with modifications to both the lipophilic tail and hydrophilic head. Scale bar is 50 μm.

The loading properties of twelve of the fourteen FM1-43 derivatives compounds (those in Groups II-VI) were also tested in mouse cochlear cultures. Each compound was tested at a single concentration of 0.3 µM. Images were taken at 5, 10 and 15 min after application of the compound and loading was compared relative to that of the parent compound 13550. Representative images are shown in Figure 6. Generally the results were similar to those observed in zebrafish. For example, compound 14885 failed to load (Figure 6B), compounds 14957 (Figure 6C) and 13668 (Figure 6D) loaded better than 13550 (Figure 6A), and a reduction in hair-cell specificity for dye loading was observed with compounds 13695, 14957, 13668 and 28552 (Figures 6B–D, F respectively). Compound 13669 did, however, appear to load more efficiently into the hair cells of mouse cochlear cultures (Figure 6D) relative to those of zebrafish neuromasts (Figure 5E).

**FIGURE 7:**
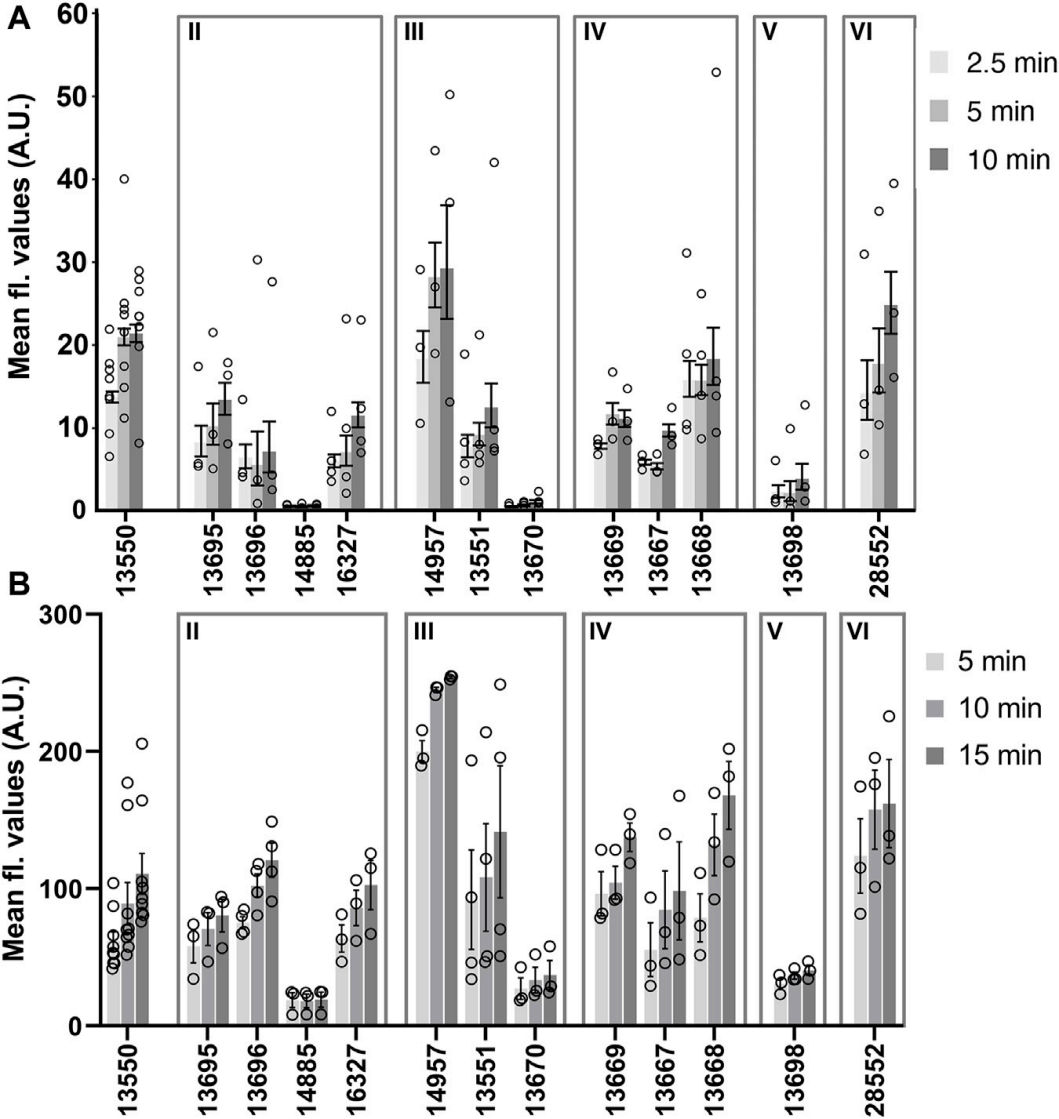
Loading levels observed with FM1-43 and its derivatives in zebrafish lateral line neuromasts **(A)** and mouse cochlear outer hair cells **(B)**. **(A)** Background corrected fluorescence values measured in neuromasts after loading with 3 μM of FM1-43 or its derivatives for 2.5, 5, and 10 min. Each circle represents the average loading for neuromast 4 from individual fish. N numbers are as follows: FM1-43 (13550) *n* = 8, 13695 *n* = 3, 13696 *n* = 3, 14885 *n* = 3, 16327 *n* = 4, 14957 *n* = 3, 13551 *n* = 4, 13670 *n* = 4, 13669 *n* = 3, 13667 *n* = 3, 13668 *n* = 4, 13698 *n* = 3 and 28552 *n* = 3. **(B)** Fluorescence values measured in mouse OHCs after loading with FM1-43 or its derivatives for 5, 10 and 15 min. Each circle represents the mean of 10 consecutive OHCs in a cochlear culture. N numbers (cochlear cultures) are as follows: FM1-43 (13550) *n* = 9, 13695 *n* = 3, 13696 *n* = 4, 14885 *n* = 3, 16327 *n* = 3, 14957 *n* = 3, 13551 *n* = 4, 13670 *n* = 3, 13669 *n* = 3, 13667 *n* = 3, 13668 *n* = 3, 13698 *n* = 3 and 28552 *n* = 3. Values (grey bars) are means and error bars are SEM.

A comparison of the mean fluorescence intensity values measured in zebrafish neuromasts and mouse cochlear cultures for the 12 derivatives that were tested in both model systems is shown in Figures 7A, B. Whilst these data further show that the derivatives exhibit generally similar dye-loading properties in both the fish and the mouse sensory organs, the statistical analysis of dye loading data provided in the Supplementary Material (Supplemental Methods, Results and Figures), reveals there may be differences between the way in which some, but not all, of the compounds behave in the two different assays.

### Testing for potential otoprotective properties in mouse cochlear cultures

The parent compound 13550 and its derivatives were also tested in cochlear cultures to determine if they can protect mammalian hair cells against the damage induced by exposure to 5 µM gentamicin, a concentration of the aminoglycoside antibiotic which simulates the concentration in the endolymph responsible for causing ototoxicity during systemic application (Tran Ba Huy et al., 1981). All compounds were initially screened at a concentration of 5 µM over 48 h in the presence of 5 µM of gentamicin (Supplementary Figure S2). Under these conditions parent compound 13550 was found to be highly toxic, causing widespread death of both sensory and non-supporting cells, whilst 5 µM gentamicin alone caused the selective death of hair cells (Supplementary Figure S2A). Seven of the derivatives, one from Group II (16327), two from Group III (14957 and 13551), all three from Group IV (13669, 13667 and 13668) and the Group VI compound (28552) were found to be generally cytotoxic to the entire cochlear culture when co-administered with gentamicin, killing both sensory hair cells and non-sensory supporting cells (Supplementary Figures S2C–E, G). The two Group I derivatives (13547 and 13248) were not protective, nor were Groups II compounds 13696 and 14885 (Supplementary Figures S2B, C). In contrast, three compounds provided protection from the gentamicin-induced loss of OHCs. These were Group II compound 13695, Group III compound 13670 and Group V compound 13698 (Supplementary Figures S2C, D, F).

**FIGURE 8:**
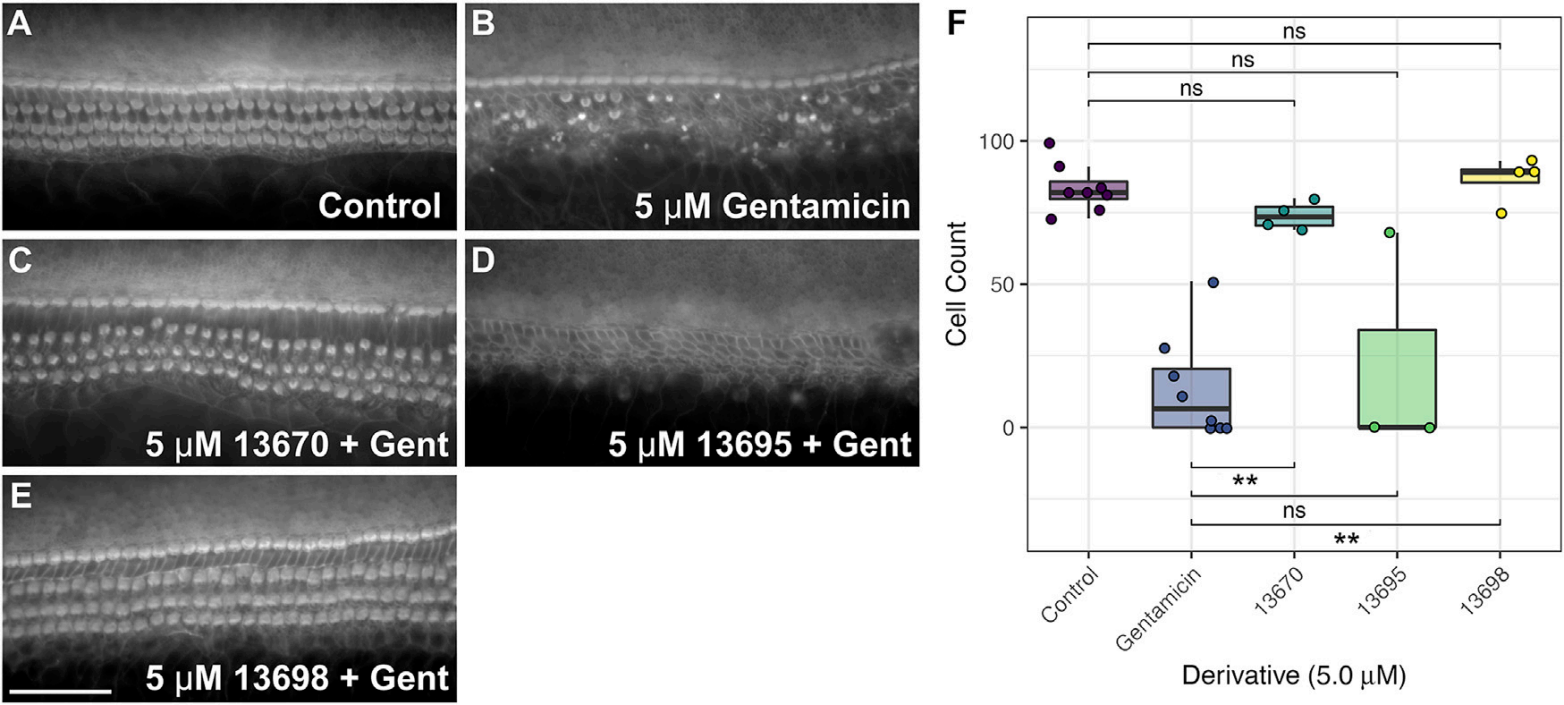
FM1-43 derivatives 13670 and 13698 protect hair cells from gentamicin-induced hair cell death at 5 μM. Cochlear cultures were treated with **(A)** 0.5% DMSO, **(B)** 5 μM gentamicin +0.5% DMSO, and **(C–E)** 5 μM gentamicin +5 μM of either 13670 **(C)**, 13695 **(D)** or 13698 **(E)**. Scale bar = 50 μm. **(F)** Box-whisker plots showing number of hair cells remaining in a 221 μm length of the mid-basal region of mouse cochlear cultures treated with either DMSO (control), 5 μM of gentamicin, or 5 μM of one of three FM1-43 derivatives +5 µM gentamicin for 48 h. Filled circles show raw data points, boxes span the interquartile range (IQR) with midlines showing median values. Tukey whiskers span and additional 1.5 x IQR. *p* ≤ 0.01 ** (for exact values see Supplementary Tables S3A, B).

The three compounds that provided protection at 5 µM were re-screened to confirm their protective abilities (Figures 8A–F). Compound 13695 failed to protect on these subsequent trials (Figures 8D, F, Supplementary Tables S3A, B) whilst compounds 13670 and 13698 were successful in providing complete protection (Figures 8C, E, F, Supplementary Tables S3A, B). Although compounds 13670 (Figure 8C) and 13698 (Figure 8E) were both subsequently and consistently protective, both caused some degree of hair bundle disruption in all three independent trials.

Given the toxic effect of most of the compounds at 5 μM, the concentration was reduced to 0.5 µM (Supplementary Figures S3A–G). At this concentration general cytotoxicity in the presence of gentamicin was not observed for any of the compounds including 13550. However, 13550 and nine of its derivatives, Group I compounds 13547 and 13248, Group II compounds 13695, 13696 and 14885, Group III compound 14957, and Group IV compounds 13669, 13667 and 13668 were not protective (Supplementary Figures S3B–E). Four were found to provide full protection in this initial screen at 0.5 µM; Group II compound 16327 (Supplementary Figure S3C), Group III compounds 13551 and 13670 (Supplementary Figure S3D), and Group VI compound 28552 (Supplementary Figure S3G).

**FIGURE 9:**
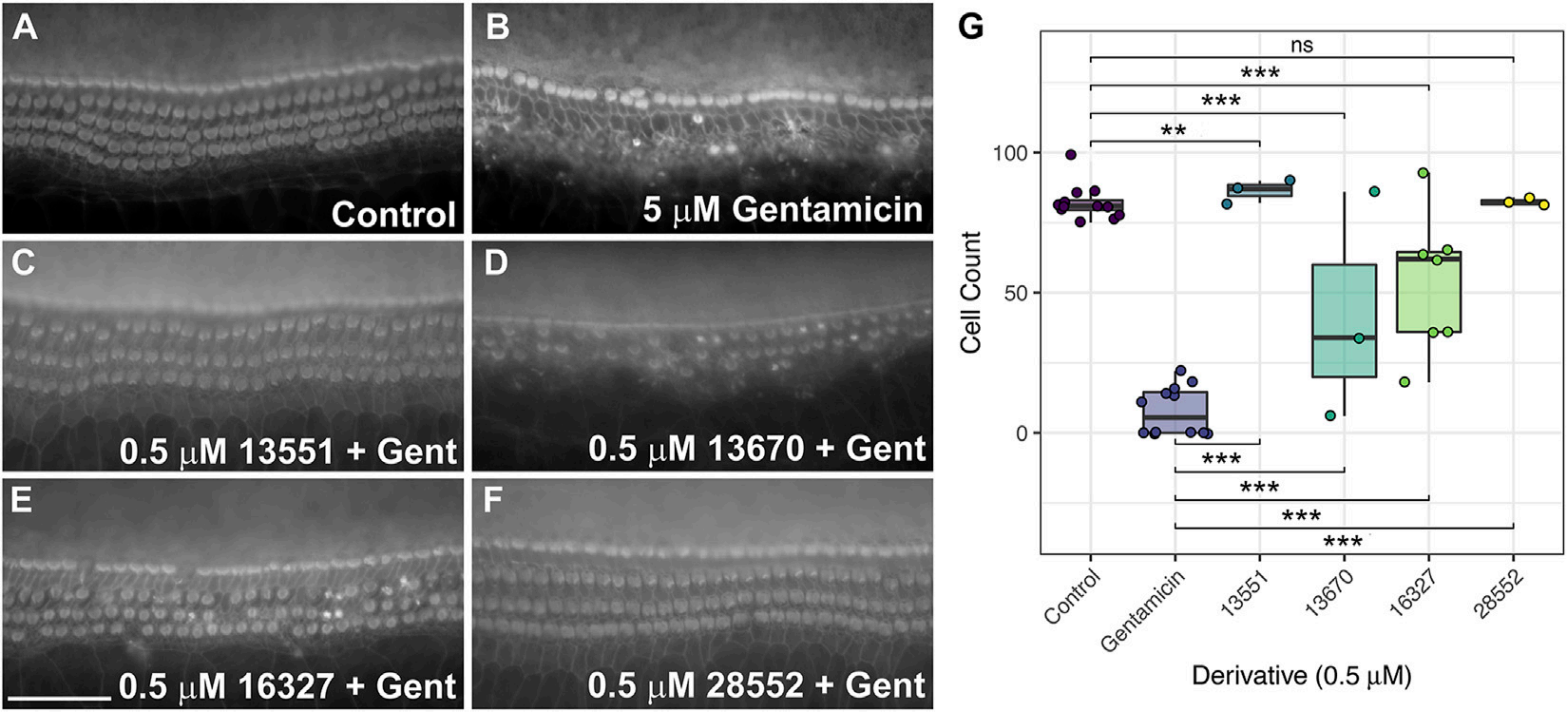
FM1-43 derivatives 13551 and 28552 protect hair cells from gentamicin-induced hair cell death at 0.5 μM. Cochlear cultures were treated with **(A)** 0.5% DMSO, **(B)** 5 μM gentamicin +0.5% DMSO and **(C–F)** 5 μM gentamicin +0.5 μM of either 13551 **(C)**, 13670 **(D)**, 16327 **(E)** or 28552 **(F)**. Scale bar is 50 μm. **(G)** Box-whisker plots of mammalian hair cell counts following exposure to gentamicin for 48 h, testing levels of protection afforded by the four FM1-43 derivatives. Filled circles show raw data points, boxes span the interquartile range (IQR) with midlines showing median values. Tukey whiskers span and additional 1.5 x IQR. *p* ≤ 0.001 ***, *p* ≤ 0.01 ** (For exact values see Supplementary Tables S3C, D).

The four that proved fully protective in the initial screen at 0.5 µM were re-screened on two or more trials to confirm their protective abilities. Two (13670 and 16327) did not provide full protection in any of the subsequent trials (Figures 9D, E, G, Supplementary Tables S3C, D). The two other derivatives, 13551 (Figures 9C, G, Supplementary Tables S3C, D) and 28552 (Figures 9F, G, Supplementary Tables S3C, D), consistently provided complete protection across all three independent trials (Figure 9G). Both derivatives, however, caused some degree of bundle disruption in all trials, possibly due to the presence of gentamicin or the combination of gentamicin and compound.

## Discussion

The fluorescent dye FM1-43 is a permeant blocker of the auditory hair cell’s MET channel (Gale et al., 2001), providing a useful tool for studying MET channel function. Using novel FM1-43 derivatives synthesised in-house and two model biological systems (organotypic mouse cochlear cultures and zebrafish larvae), we assessed which aspects of its chemical structure are fundamental to its interaction with the MET channel. Additionally, we compared the ability of the derivatives to load into hair cells and their ability to protect OHCs against cell death caused by the ototoxic aminoglycoside antibiotic gentamicin. The results of our study are summarised in Table 1 which shows, for each compound, its basic physiochemical properties, whether or not it blocks the MET channel in mouse cochlear hair cells, how well or otherwise it loads into zebrafish lateral line and mouse cochlear OHCs relative to the parent compound FM1-43 (13550), and if it affords any protection against gentamicin-induced hair-cell loss in mouse cochlear cultures.

**TABLE 1:** Summary of Physicochemical Properties and Structure-Activity Relationships of FM1-43 and its Derivatives.

Group	Compound	Mol. Weight (free base)	cLogP	No. Of charges	MET block	Cell loading		Otoprotection	
						Zebrafish	Cochlea	5 μM	0.5 μM
FM1-43	13550	452	−1.11	2	Yes	Yes	Yes	T	No
I	13547	308	5.85	0	No	Yes ↓	NT	No	No
	13248	278	−4.66	2	No	NT	NT	No	No
II	13695	564	2.45	2	Yes	Yes	Yes	No	No
	13696	644	0.76	2	Yes°	Yes ↓	Yes	No	No
	14885	512	−3.79	2	No	No	No	No	No
	16327	900	5.39	2	Yes	Yes ↓	Yes	T	No
III	14957	352	2.79	1	Yes	Yes ↑	Yes ↑	T	No
	13551	424	2.41	1	Yes	Yes ↓	Yes ↑	T	Yes
	13670	396	0.73	Zwitterionic	Yes	Yes ↓	Yes ↓	Yes	No
IV	13669	466	−0.59	2	Yes	Yes ↓	Yes ↑	T	No
	13667	480	−0.14	2	Yes	Yes ↓	Yes	T	No
	13668	494	0.3	2	Yes	Yes	Yes	T	No
V	13698	659	3.7	2	*	Yes ↓	Yes ↓	Yes	No
VI	28552	636	4.28	1	Yes	Yes ↓	Yes ↑	T	Yes

° = tested at 3 µM (all other compounds were tested at 10 µM), * = could not be tested due to precipitation during superfusion, T = cytotoxic, NT = not tested, Yes↓ = and Yes↑ = significantly less (*p* < 0.01) and significantly more (*p* < 0.01) loading respectively than that observed with 13550 at 3 μM at the 5 min time point in zebrafish (Supplementary Table S1), and in mouse cochlear cultures with 0.3 μM at the 5 min time point (Figure 7).

### Structure-activity relationship of FM1-43 derivatives

Overall, the results reveal how the charged hydrophilic head and the hydrophobic tail are both required to enable FM1-43 to interact with the MET channel, and how the efficacy of the block and loading into the hair cell can be modulated. Of the thirteen derivatives tested for their ability to block the MET current in patch-clamped mouse OHCs at different membrane potentials, three failed to block the current. These were the two truncated Group I derivatives, one without the hydrophobic tail (13248) and the other lacking the hydrophilic head region (13547), and the Group II derivative 14885 in which the lipophilicity of the tail region was considerably reduced by replacing the alkyl chains with carboxylic ester moieties. A fourth compound, the zwitterionic Group III compound 13670 in which the quaternary nitrogen was replaced by an alkyl acid that is negatively charged at physiological pH, only provided a weak block of the MET current at negative membrane potentials. Together, the data from these four compounds are sufficient to reveal the importance and necessity of both a cationic head group and a lipophilic tail for MET channel block by FM1-43.

Of the other derivatives, Group II tail derivative 13696 with a phenyl ring at the end of each alkyl tail, proved to be the most effective MET channel blocker of all tested, providing >90% block at a concentration of 3 μM and a membrane potential of −64 mV, a value near that of the resting potential of OHCs in mouse cochlear cultures (Marcotti et al., 1999; Marcotti and Kros, 1999). Other derivatives producing a similar level of block (>80% at −64 mV), but at a slightly higher concentration (10 μM) all had modifications of the head region. These were the two Group III compounds, 14957 and 13551, in which the terminal quaternary nitrogen is replaced by an alkyl and an ester group respectively, and Group IV compounds 13669 and 13667 in which the spacer between the two positively charged nitrogens of the head region is increased from a start value of 3 to 4 and 5 carbons, respectively. It would therefore appear that either removing one of the two positively charged N atoms in the head group, or increasing the distance between them, or increasing the lipophilicity of the alkyl tail can augment block of the MET current compared to FM1-43 (∼30% block at −64 mV), providing further evidence that both moieties of FM1-43 (the head and the tail) cooperate to reduce ion flow through the MET channel.

All of the FM1-43 derivatives that were found to block the hair-cell MET current behaved as permeant blockers, showing a release of block when the membrane potential was shifted to either hyper- or depolarized levels. Whilst it is not possible to calculate permeation rates through the MET channel for these compounds, loading into the cell through this route can be assessed using fluorescence microscopy. The results obtained from the dye loading assays performed with the mouse cochlear cultures are, for the most part, as expected from the electrophysiological data discussed above. For example, Group II compound 14485 with the hydrophilic tail and Group III compound 13670 with the zwitterionic head, compounds that provided no or very little block at negative potentials respectively, exhibited no or very weak labelling. Also, compound 14957, a Group III compound that provided a very strong block of the MET channel (∼90% block at −64 mV) with steep release of block at more hyperpolarised potentials (indicative of the compound permeating through the channel), loaded rapidly and provided strong labelling of hair cells (relative to 13550) at early time points. The loading assay may not, however, be able detect subtle differences in how a compound interacts with the MET channel. For example, Group II compounds 13695, 13696, and 16327 all vary in how well they block the MET channel and the extent to which the block is released at increasingly hyperpolarised potentials relative to 13550, but show similar labelling to 13550 as a function of time in mouse cochlear cultures. It should, however, be noted that the labelling intensity observed may not directly relate to dye loading as the quantum yield of the derivatives may be affected by the structural modifications. The fluorescence assays did, nonetheless, reveal that some of the derivatives had staining properties that were less selective for hair cells than the parent compound, as they also loaded into supporting cells located in and around the sensory organs, both in zebrafish neuromasts and mouse cochlear cultures. This was particularly evident for Group VI compound 28552 (cLogP 4.28) and compounds that were more lipophilic than other members of their group (e.g., Group II compounds 13695 and 16327, Group III compound 14957 and Group IV compound 13668), and may therefore be a direct consequence of this property.

### Limited potential of FM1-43 derivatives for protection from aminoglycoside ototoxicity

Although it has been shown previously that 30 µM FM1-43 can reduce the acute morphological effects seen in the hair cells of mouse cochlear cultures caused by short-term exposure (1 h) to a high concentration (1 mM) of the aminoglycoside antibiotic neomycin at room temperature (Gale et al., 2001), the results of the current study revealed that long-term exposure (48 h) to 5 µM FM1-43 in the presence of 5 µM gentamicin at 37°C leads to widespread cell death throughout the culture. Under similar conditions, 5 µM gentamicin alone selectively kills OHCs in the basal, high-frequency end of the cochlear explant. Of all 14 FM1-43 derivatives tested at 5 µM in the long-term, low-dose cochlear culture assay, eight were, like 13550, generally cytotoxic to varying degrees whilst two protected the OHCs. These were the Group III zwitterion 13670 and the Group V bis-derivative (13698). Unfortunately, neither derivative provided full protection when re-tested at a ten-fold lower concentration (0.5 µM), a concentration at which none of the compounds, including the parent molecule 13550, were generally cytotoxic. Nonetheless, two other otoprotective “hits” emerged, the single-charged Group III derivative 13551 and the Group VI derivative 28552 in which two modifications, a bulky hydrophilic tail and a singly charged head, are merged into one molecule. Both compounds behave as permeant blockers of the hair-cell MET channel, with 13551 being the most efficient blocker of all tested at −60 mV, and both exhibited a highly significant (*p* < 0.001, Supplementary Table S2) increase in loading relative to 13550 in mouse cochlear cultures. It is, however, still questionable if these two compounds merit further investigation as (1) it remains unknown if these molecules have any effect on systems other than the hair-cell MET channel and (2) some signs of hair bundle disruption were evident in the protected hair cells. The latter, though, may not be a problem as short-term pharmacological block of MET channel activity is known to cause changes in the shape and size of stereocilia that are, on drug washout, reversible (Vélez-Ortega et al., 2017; Krey et al., 2020). Whether or not the effects of the FM1-43 derivatives on hair bundle structure can also be reversed is an outstanding question that needs to be addressed.

### FM1-43 and aminoglycosides may enter the MET channel via separate pathways

A final aspect to be considered is how FM1-43 and the aminoglycoside antibiotics, both of which behave as permeant blockers of the hair-cell MET channel from an electrophysiological perspective, selectively enter into sensory hair cells via this ion channel. Whilst the aminoglycosides are hydrophilic polycations, FM1-43 has a cationic head group and a lipophilic tail both of which, as shown in this study, are required for it to block the MET channel and enter the hair cell.

There is now considerable evidence that the ion-conduction pathway of the hair-cell MET complex is formed by transmembrane channel like protein TMC1 (and/or TMC2 in immature cochlear hair cells and vestibular hair cells), possibly in close association with the transmembrane protein of the inner ear, TMIE (Cunningham and Müller, 2019; Cunningham et al., 2020). TMC1 is related to TMEM16A (a calcium-activated chloride channel) and TMEM16F (a calcium-activated lipid scramblase) and, on the basis of cryo-EM and X-ray structures for these two proteins respectively, it was proposed that TMC1 was a dimer composed of two subunits each with 10 transmembrane (TM) domains, with TM domains 4–7 of each subunit forming a separate ion-conducting, lipid membrane-facing cavity (Ballesteros et al., 2018; Pan et al., 2018). Important evidence for the involvement of TM domains 4–7 in cation conduction was provided by cysteine substitution experiments (Pan et al., 2018) and data derived from a variety of mouse mutants which have identified key amino acid residues in the cation permeation pathway (Beurg et al., 2021b; Beurg et al., 2021a; Beurg et al., 2019).

More recently, single particle cryo-EM (Jeong et al., 2022) has been used to resolve the structure of a native mechano-sensory complex purified from *C. elegans* that is comprised of two copies each of TMC1, TMIE, and CALM1, with the latter being an invertebrate protein related to the calcium and integrin binding protein CIB2/3, an essential non-transmembrane protein that is closely associated with the cytoplasmic face the hair-cell MET channel complex (Giese et al., 2017; Michel et al., 2017; Wang et al., 2017). The cryo-structure of this complex reveals that the two TMC1 subunits form a symmetrical dimer, with TM domains 4–8 of each TMC1 monomer forming an apparently closed ion conduction pathway at opposing, outwardly facing edges of the dimer, adjacent to each of which lies the single pass TM helix of each TMIE subunit. Although the TM domain of each TMIE subunit lies adjacent to two of the TM domains in TMC1 that contribute to the ion conduction pathway (TM6 and TM8), direct protein-protein contacts between the TM helices of TMIE and TMC1 within the plane of the membrane are very limited. Instead, there is an intramembranous cavity within which lipids (including the palmitoylated acyl group associated with cysteine 44 of the TMIE TM helix) form bridges between the two proteins. Based on these findings, it has been suggested that TMIE and adjacent or associated lipids may play a key role in sensing and transmitting changes in membrane tension to the ion conducting pore of TMC1. Furthermore, it has been speculated that the lipid-filled space between TMC1 and TMIE, and/or other hydrophobic crevices between the TM domains of TMC1 that form the ion conduction pathway, may allow larger molecules like FM1-43 to enter hair cells via the MET channel complex (Jeong et al., 2022).

Whether or not the lipid-filled cavity between TMIE and TMC1 provides a strictly alternative (i.e., spatially distinct) pathway for FM1-43 entry into hair cells to the cation conduction pore, as in the out-of-the-groove model proposed to explain how ions and lipids both can pass through lipid scramblases (Kalienkova et al., 2021), remains to be determined. Nonetheless, FM1-43 is a permeant blocker of the vertebrate hair-cell MET channel that can, unlike other MET channel blockers such as dihydrostreptomycin, amiloride and related compounds, and d-tubocurarine (Rüsch et al., 1994; Glowatzki et al., 1997; Marcotti et al., 2005; Alharazneh et al., 2011; Kirkwood et al., 2017) reside in both the closed as well as the open state of the channel (Gale et al., 2001; van Netten and Kros, 2007). Furthermore, as an elongated molecule (length about 2.2 nm) with an end-on diameter of ∼0.8 nm, FM1-43 may not be able to reside deep within the cation permeation pore of TMC1, the narrowest constrictions of which have been estimated to have a diameter of ∼0.2 nm in the closed channel (Jeong et al., 2022). Moreover, with a Hill coefficient of up to 2, two FM1-43 molecules may simultaneously reside in the channel. If FM1-43 indeed resides in a separate groove and not in the pore, the question arises how it blocks MET currents, although the presence of FM1-43 in a spatially distinct region may physically prevent the pore from reaching its much wider open configuration [estimated to be at least 1.2 nm in diameter (Farris et al., 2004)]. It therefore seems quite likely that FM1-43 may enter hair cells via the lipid-rich cavity present between the TM domain of TMIE and TM domains 6 and 8 of TMC as proposed by Jeong et al. (2022). This possibility is further supported by the recent observations that TMC1 may be able to act, in response to MET channel block or application of BAPTA-AM (both of which should reduce intracellular free calcium within the hair cell), as a phospholipid scramblase that is, somewhat unexpectedly and atypically, inactivated by calcium (Ballesteros and Swartz, 2022).

**FIGURE 10:**
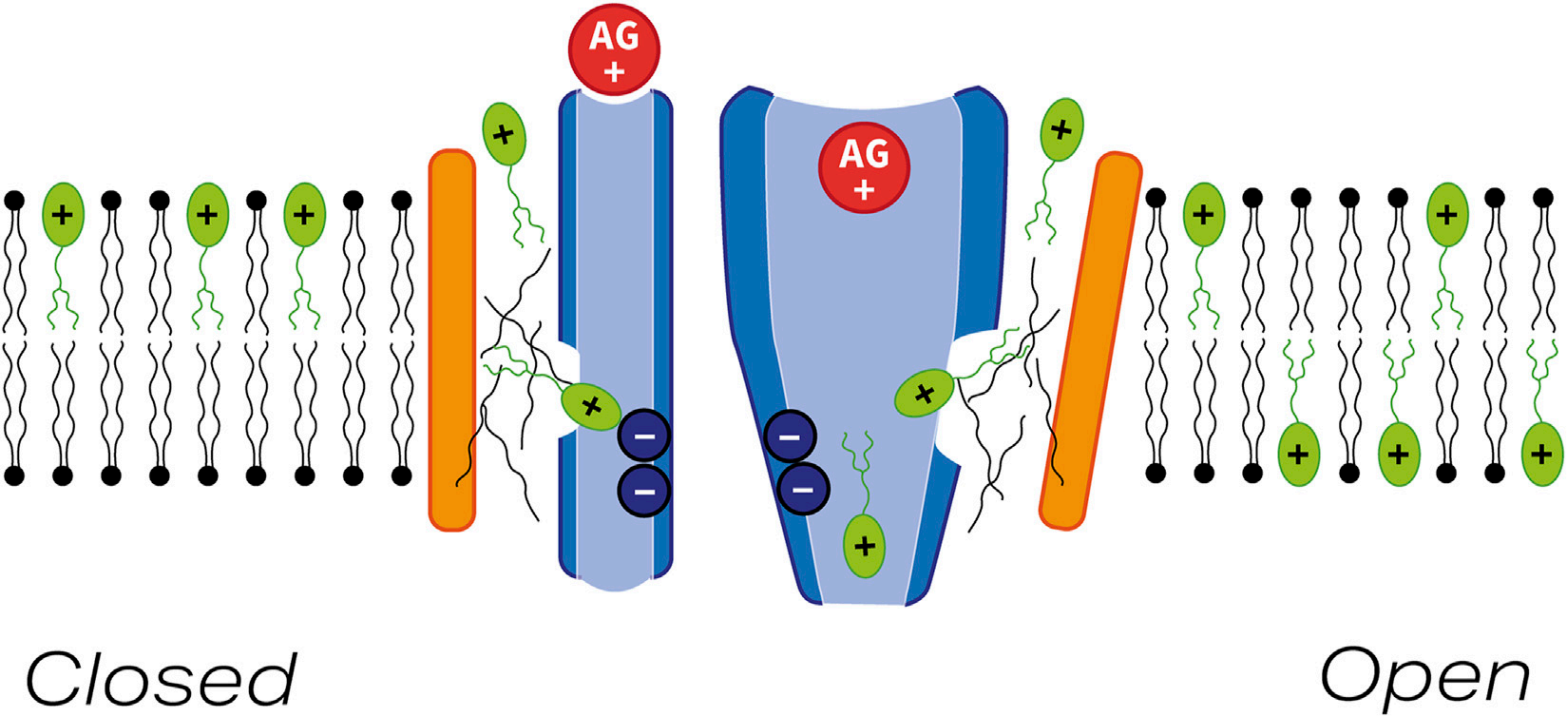
Model indicating possible pathway for FM1-43 entry into hair cells via the MET channel. Schematic diagram showing pore-forming region of TMC1 (blue) and the closely-associated transmembrane helix of TMIE (orange) with the MET channel in either the closed (left) or open (right) state. FM1-43 and an aminoglycoside are shown in green and red respectively. Thin black lines depict lipid chains. A small region of communication between the lipid-rich region lying between TMIE and the pore-forming region of TMC1 may allow FM1-43 to block the channel in both the closed and the open states. In the mechanically activated open state FM1-43 and aminoglycoside can both enter the hair cell via the MET channel.

Whilst a strictly alternative, lipid-rich pathway for FM1-43 entry into hair cells is an attractive concept, there are other aspects of FM1-43 mediated MET-channel block and the loading of FM1-43 into hair cells that need to be considered. Firstly, a cationic, positively-charged head group is required for MET-channel block, and secondly, although FM1-43 can reside in the closed state of the channel, the channel has to be open for dye loading to occur, as shown by experiments in which loading fails following destruction of the tip links with calcium chelators (Gale et al., 2001; Alagramam et al., 2011; Geng et al., 2012). Thirdly, dominant missense mutations (T416K and D569N) of residues located in TM domains 4 and 7 of TMC1 lying close to the cytoplasmic side of the cation pore have been shown to reduce FM1-43 accumulation in mouse cochlear hair cells (Beurg et al., 2021b). An alternative possibility is that the cation conducting pore, which is assumed to be the route that is exclusively used by the aminoglycosides to access the hair-cell cytosol, and the lipid-rich FM1-43 entry pathway are not entirely separate entities and may communicate with each other at some point through the plane/depth of the membrane (see Figure 10). In this model, the aminoglycosides and FM1-43 would enter the channel via separate routes, but exit it into the cytosol via the same shared pathway. Increasing lipophilicity of the acyl tail of FM1-43 may enhance permeation to the site of communication or reduce the transmission of gating forces to the channel, and reducing charge on the head group or altering the distance between the two positively charged quarternary nitrogens may influence interactions with negatively-charged sites in parts of the pore nearer the region where cations exit into the cytoplasm.

In conclusion, a strictly alternative pathway for FM1-43 entry into hair cells may not be required. Nonetheless it would be invaluable to determine exactly where FM1-43, the ototoxic aminoglycoside antibiotics, and other lipophilic blockers of the hair-cell MET complex (e.g., those derived from amiloride described by Rüsch and colleagues in 1994 and the lipophilic MET channel-blocking otoprotectants discovered by Kenyon et al., 2021) bind within or relative to the ion conduction pathway of TMC1 (Rüsch et al., 1994; Kenyon et al., 2021).

## Data Availability

All relevant data are included within the results section of the paper and in the Supplementary Material.
