# Supplementary material for: Charge and lipophilicity are required for effective block of the hair-cell mechano-electrical transducer channel by FM1-43 and its derivatives
Source: Front Cell Dev Biol. 2023 Oct 10;11:1247324. doi: 10.3389/fcell.2023.1247324 (PMC10601989; doi:10.3389/fcell.2023.1247324)
Supplement: Supplementary file 1 [file DataSheet2.pdf]

Supplemental Chemical Synthesis

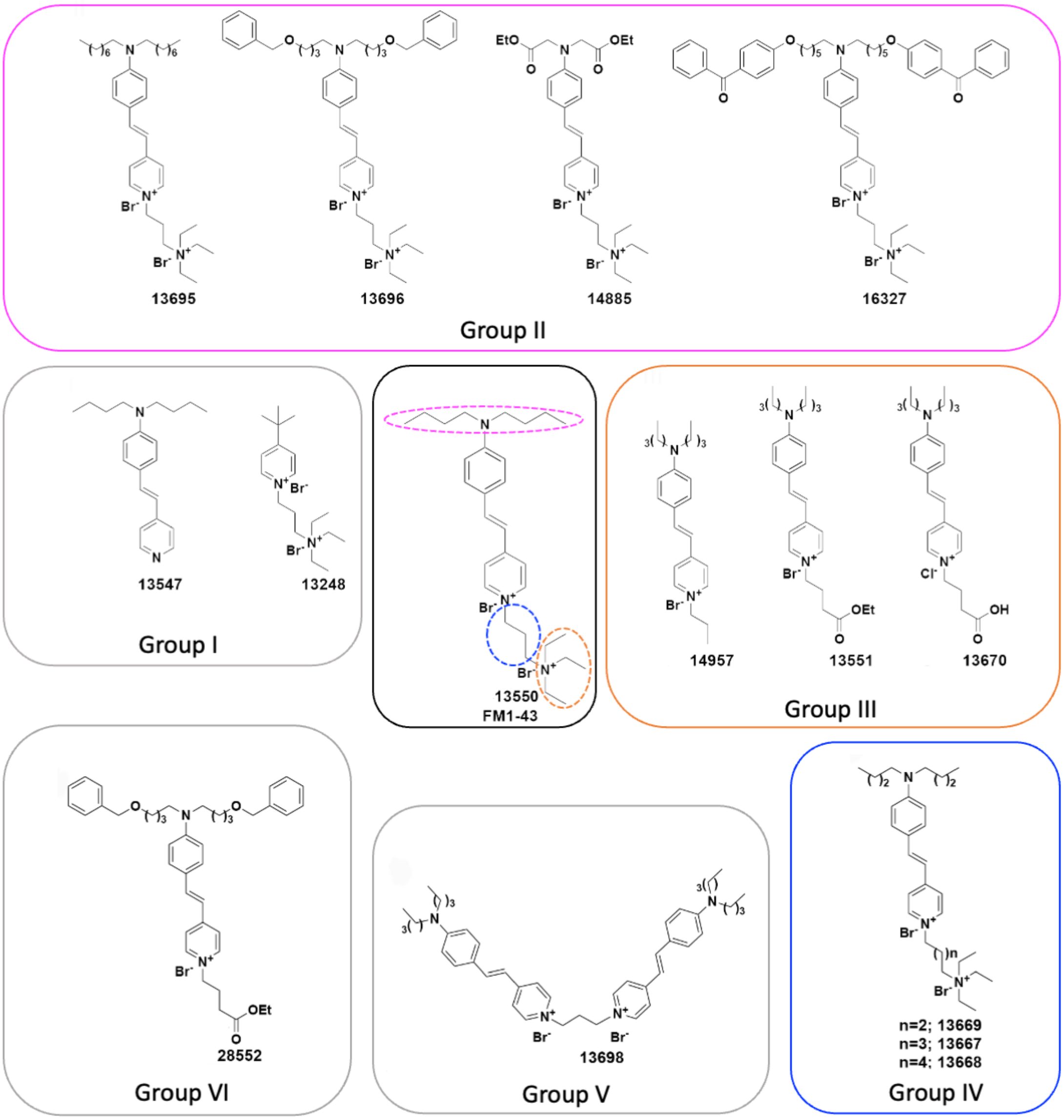

Compounds used in the study are shown above and are highlighted in yellow in the text below.

All commercial reagents were purchased from Sigma-Aldrich, Alfa Aesar, Apollo Scientific, Fluorochem or Tokyo Chemical Industry and of the highest available purity. Unless otherwise stated, chemicals were used as supplied without further purification. Anhydrous solvents were purchased from Acros (AcroSeal) or Sigma-Aldrich (SureSeal) and were stored under nitrogen. Petroleum ether refers to the fraction with a boiling point between 40 and 60 °C. Thin-layer chromatography: precoated aluminum-backed plates (60 F254, 0.2 mm thickness, Merck) were visualized under both short- and longwave UV light (254 and 366 nm). Flash column chromatography was carried out using commercial prepacked columns from Biotage, Isco, Grace, or filled with Merck silica gel 60 (40−63 µm) or C18 silica on an ISCO Combiflash Rf or a Biotage Isolera Prime. HPLC purification was performed on an Agilent 1100 series HPLC spectrometer, using a Phenomenex Luna 10 µm C18 150 mm × 15 mm column, eluted using water and acetonitrile at 15 mL/min and detected at 254 nm.

Proton nuclear magnetic resonance spectra were recorded at 500 or 600 MHz on a Varian VNMRS 500 MHz or Varian VNMRS 600 MHz spectrometers, respectively (at 30 °C), using residual isotopic solvent (CHCl3, δ = 7.27 ppm, DMSO δ = 2.50 ppm) as an internal reference. Chemical shifts are quoted in parts per million (ppm). Coupling constants (J) are recorded in hertz (Hz). The following abbreviations are used in the assignment of NMR signals: s (singlet), d (doublet), t (triplet), q (quartet), qn (quintet), m (multiplet), bs (broad singlet), dd (doublet of doublet), and dt (doublet of triplet). Carbon nuclear magnetic resonance spectra were recorded at 125 or 151 MHz on Varian 500 or 600 MHz spectrometers, respectively, and are proton-decoupled, using residual isotopic solvent (CHCl3, δ = 77.00 ppm, DMSO δ = 39.52 ppm) as an internal reference.

LCMS data were recorded on a Waters 2695 HPLC using a Waters 2487 UV detector and a Thermo LCQ ESI-MS. Samples were eluted through a Phenomenex Lunar 3 µm C18 50 mm × 4.6 mm column, using water and acetonitrile acidified by 0.1% formic acid at 1 mL/ min and detected at 254 nm. The following methods were used: method 1: water (+0.1% formic acid)/acetonitrile (+0.1% formic acid) = from 65/35 to 10/90 in 3.5 min, then isocratic 10/90 0.4 min, then from 10/90 to 65/35 in 0.1 min; method 2: water (+0.1% formic acid)/acetonitrile (+0.1% formic acid) = from 70/30 to 10/90 in 5 min, then isocratic 10/90 1.0 min, then from 10/90 to 70/30 in 0.5 min, and then isocratic 70/30 for 0.5 min.

LCMS (MDAP): LCMS data were recorded on a Shimatzu Prominence Series coupled to a LCMS-2020 ESI and APCI mass spectrometer. Samples were eluted through a Phenomenex Gemini 5µ C18 110A 250 mm × 4.6 mm column, using water and acetonitrile acidified by 0.1% formic acid at 1 mL/min and detected at 254 nm. The following method, marked as method 3, was used: water (+0.1% formic acid)/acetonitrile (+0.1% formic acid) = isocratic 95/5 1 min, then from 95/5 to 5/95 in 20 min, then isocratic 5/95 for 4 min, and then from 5/95 to 70/30 in 5 min. Physicochemical properties were calculated using MarvinSketch 16.8.15.0 by ChemAxon (https://www.chemaxon.com). Compound purity was assured by a combination of high-field multinuclear NMR (H, C) and HPLC; purity by the later was always >95%.

Scheme 1

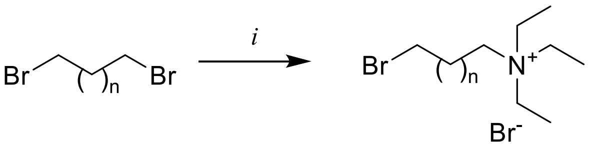

n=1 N2009-1; 26%
n=2 N2009-2; 17%
n= 3 N2009-9, 21%
n=4 N2009-10, 13%

Reagents and conditions: *i*: triethylamine, appropriate dibromoalkane, rt, 24 h.

Synthesis of 3-bromopropyl(triethyl)ammonium bromide. **N2009-1.**

A stirring solution of triethylamine (5.0 mL, 35.87 mmol) and 1,3-dibromopropane (18.21 mL, 179.37 mmol) in tetrahydrofuran (15 mL) was stirred at room temperature for 24 h. After this period, diethyl ether was added and the suspension was filtered to give the desired compound as an hygroscopic pale brown solid in two different crops (2.80 g, 26%). ^{1}H NMR (DMSO 500 MHz): δ 3.66-3.56 (m, 2H), 3.33-3.20 (m, 8H), 2.22-2.11 (m, 2H), 1.18 (t, J = 7.2 Hz, 9H). ^{13}C NMR (DMSO 126 MHz): δ 55.15, 52.73, 35.10, 32.92, 31.16, 24.95, 7.63. LCMS: 7 min method: RT: 0.55 min, M-Br$^-$: 222.24, 224.24.

Synthesis of 4-bromobutyl(triethyl)ammonium bromide. **N2009-2.**

A solution of triethylamine (5.0 mL, 35.87 mmol) and 1,4-dibromobutane (21.42 mL, 179.37 mmol) in tetrahydrofuran (15 mL) was stirred at room temperature for 24 h. After this period, diethyl ether was added and the suspension was filtered to give the desired compound as an hygroscopic pale brown solid (2.00 g, 17%). ^{1}H NMR (DMSO 500 MHz): δ 3.60 (t, J = 6.6 Hz, 2H), 3.24 (q, J = 7.2 Hz, 6H), 3.20-3.16 (m, 2H), 1.90-1.82 (m, 2H), 1.76-1.67 (m, 2H), 1.23-1.13 (m, 9H). ^{13}C NMR (DMSO 126 MHz): δ 55.56, 52.64, 46.16, 34.43, 29.49, 20.26, 7.67. LCMS: 7 min method: RT: 0.54 min, M-Br$^-$: 236.15, 238.27.

Synthesis of 5-bromopentyl(triethyl)ammonium bromide. **N2009-9.**

A stirring solution of triethylamine (4.0 mL, 28.7mmol) and 1,5-dibromopentane (19.55 mL, 143.5 mmol) in tetrahydrofuran (10 mL) was stirred at room temperature for 24 h. After this period, diethyl ether was added and the suspension was filtered to give the desired compound (N1009-9-1) as an hygroscopic white solid (2.10 g, 21%).^{1}H NMR (DMSO 500 MHz): δ 3.56 (t, J = 6.7 Hz, 2H), 3.24 (q, J = 7.2 Hz, 6H), 3.18-3.09 (m, 2H), 1.86 (p, J = 6.9 Hz, 2H), 1.67-1.55 (m, 2H), 1.41 (p, J = 7.6 Hz, 2H), 1.23-1.10 (m, 9H). ^{13}C NMR (DMSO 126 MHz): δ 56.37, 52.54, 35.27, 32.05, 24.97, 20.68, 7.74. LCMS: 7 min method: RT: 0.58 min, M-Br$^-$: 250.34, 252.28.

Synthesis of 6-bromohexyl(triethyl)ammonium bromide. **N2009-10.**

A stirring solution of triethylamine (3.0 mL, 21.52 mmol) and 1,6-dibromohexane (16.56 mL, 107.62 mmol) in tetrahydrofuran (8 mL) was stirred at room temperature for 24 h. After this period, diethyl ether was added and the suspension was filtered to give the desired compound (N2009-10-01) as a hygroscopic pale brown gum (1.05 g, 13%). ^{1}H NMR (DMSO 500 MHz): δ 3.53 (t, J = 6.7 Hz, 2H), 3.24

(q, J = 7.2 Hz, 6H), 3.15-3.08 (m, 2H), 1.86-1.76 (m, 2H), 1.63-1.52 (m, 2H), 1.49-1.36 (m, 2H), 1.31 (p, J = 7.5 Hz, 2H), 1.24-1.10 (m, 9H). ^{13}C NMR (DMSO 126 MHz): δ 56.50, 52.55, 35.48, 32.41, 27.52, 25.40, 21.34, 7.72. LCMS: 7min method: RT: 0.58 min, M-Br^-: 264.32, 266.26.

Scheme 2

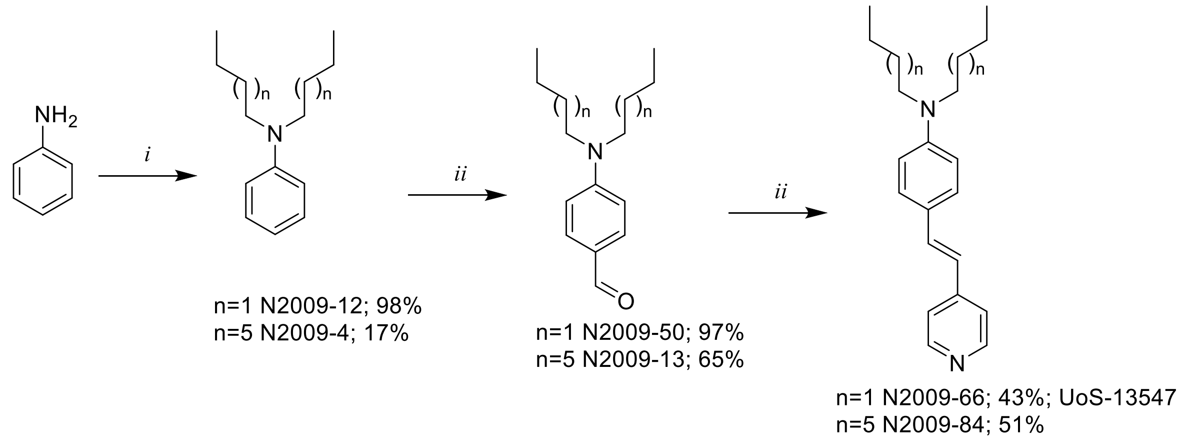

Reagents and conditions: *i*: appropriate 1-bromo-alkane, K_2CO_3, reflux, 24 h; *ii*: $POCl_3$, DMF, 5 °C, 30 min, then 80 °C, 4.5 h; *iii*: 4-methylpyridine, potassium tert-butoxide, 135 °C, 5 h;

Synthesis of N,N-dibutylaniline N2009-12.

A solution of 1-bromobutane (9.9 mL, 92.18 mmol), K_2CO_3 (12.74 g, 92.18 mmol) and aniline (2.1 mL, 23.05 mmol) was heated at reflux for 24 h. After cooling, water was added and the aqueous phase was extracted with DCM. The organic phase was washed with brine, dried over $MgSO_4$, filtered and concentrated. The residue was purified by flash column chromatography eluting with petroleum ether to give the desired compound (N2009-12-01) as a yellow oil (4.65 g, 98%). ^{1}H NMR (DMSO 600 MHz): δ 7.12-7.07 (m, 2H), 6.58 (d, J = 8.2 Hz 2H), 6.50 (t, J = 7.4 Hz, 1H), 3.25-3.18 (m, 4H), 1.50-1.41 (m, 4H), 1.33-1.25 (m, 4H), 0.89 (t, J = 7.4 Hz, 6H). LCMS: 10 min method, RT 6.80, M+H+: 206.14.

Synthesis of N,N-dioctylaniline N2009-4.

A solution of aniline (2.0 mL, 21.95 mmol) and 1-bromooctane (15.14 mL, 87.79 mmol) was heated at 150 °C for 48 h. After cooling, the reaction mixture was quenched with an excess of 1N NaOH and the aqueous phase was extracted with diethyl ether. The organic phase was dried over $MgSO_4$, filtered and concentrated. The residue was purified by flash column chromatography gradient elution of petroleum ether / ethyl acetate (100 / 0, then 90 / 10). The desired compound was further purified by flash column chromatography eluting with petroleum ether to give the desired compound as yellow oil (1.3 g, 17%). The mono alkylated derivative was also isolated (0.80 g, 16%). ^{1}H NMR (DMSO 500 MHz): δ 7.15-7.06 (m, 2H), 6.63-6.55 (m, 2H), 6.53-6.49 (m, 1H), 3.24-3.19 (m, 4H), 1.48 (t, J = 7.6 Hz, 4H), 1.33-1.20 (m, 20H), 0.88-0.82 (m, 6H). ^{13}C NMR (DMSO 126 MHz): δ 147.84, 129.48, 128.64, 111.94, 50.62, 31.74, 29.32, 29.16, 27.22, 26.93, 22.52. LCMS: 10 min method, RT 9.63, M+H+: 318.38.

Synthesis of 4-(dibutylamino)benzaldehyde N2009-50.

To cooled anhydrous N,N-dimethylformamide (55 mL) phosphorus oxychloride (4.45 mL, 47.73 mmol was added dropwise at 5 °C and the reaction mixture was stirred at the same temperature for 30 min. Then N,N-dibutylaniline (9.80 g, 47.73 mmol) in anhydrous N,N-dimethylformamide (5 mL) was added dropwise and the reaction mixture was stirred at 80 °C for 4.5 h. After cooling, the mixture was

quenched with slowly addition of ice cold water and neutralised with 5N NaOH. The aqueous phase was extracted with diethyl ether and the organic phase was washed with water (5 times), dried over $MgSO_4$, filtered and concentrated The residue was purified by flash column chromatography gradient elution of petroleum ether / ethyl acetate (100 / 0, then 100 / 0 to 20 / 80) to give the desired compound as a brown oil (10.85 g, 97%). ^{1}H NMR (DMSO 500 MHz): δ 9.62 (s, 1H), 7.68-7.59 (m, 2H), 6.73 (d, J = 8.9 Hz, 2H), 3.39-3.32 (m, 4H), 1.57-1.46 (m, 4H), 1.33 (h, J = 7.4 Hz, 4H), 0.91 (t, J = 7.4 Hz, 6H). LCMS: 7 min method: RT: 5.67 min, M+H+= 234.24

Synthesis of 4-(dioctylamino)benzaldehyde N2009-13.

To cooled N,N-dimethylformamide (8 mL) phosphorus oxychloride (0.35 mL, 3.78 mmol) was added dropwise at 5 °C and the reaction mixture was stirred at the same temperature for 30 min. Then N,N-dioctylaniline (1.20 g, 3.78 mmol) was added dropwise and the reaction mixture was stirred at 80 °C for 3 h. After cooling, the mixture was quenched with slowly addition of ice cold water and neutralised with 5N NaOH. The aqueous phase was extracted with diethyl ether and the organic phase was washed with water (5 times), dried over $MgSO_4$, filtered and concentrated. The residue was purified by flash column chromatography gradient elution of petroleum ether / ethyl acetate (100 / 0 to 50 / 50) to give the desired compound impure with the starting material which was then further purified by flash column chromatography eluting with petroleum ether / ethyl acetate (100 / 0, then 90 / 10) to give the desired compound as a brown oil (0.90 g, 65%). ^{1}H NMR (DMSO 600 MHz): δ 9.60 (s, 1H), 7.65-7.59 (m, 2H), 6.70 (d, J = 8.7 Hz, 2H), 3.36-3.31 (m, 4H), 1.51 (p, J = 6.9 Hz, 4H), 1.31-1.19 (m, 20H), 0.83 (t, J = 6.8 Hz, 6H).

Synthesis of N,N-dibutyl-4-[(E)-2-(4-pyridyl)vinyl]aniline N2009-66-7 – UoS-13547.

To a mixture of 4-methylpyridine (1.46 mL, 15.0 mmol) and 4-(dibutylamino)benzaldehyde (3.85 g, 16.5 mmol) potassium tert-butoxide (2.53 g, 22.5 mmol) was added at 135 °C every hour for 4 h. After the last addition, the reaction mixture was stirred at 135 °C for further 1 h. Then, 4-methylpyridine (0.29 mL, 3.0 mmol) and potassium tert-butoxide (2.52 g, 22.5 mmol) were added and the reaction mixture was stirred at 135 °C for further 1 h. After cooling, water was added and the precipitate was filtered. The solid was crystallised from ethanol and water to give the first crop of the desired compound as a brown solid (0.20 g, 4%). The solution was concentrated and the residue was purified by flash column chromatography gradient elution of petroleum ether / ethyl acetate (100 / 0 to 60 / 40) to give the desired compound as a brown solid (1.98 g, 43%). ^{1}H NMR (DMSO 500 MHz): δ 8.47-8.42 (m, 2H), 7.46-7.40 (m, 4H), 7.37 (d, J = 16.4 Hz, 1H), 6.87 (d, J = 16.3 Hz, 1H), 6.64 (d, J = 8.7 Hz, 2H), 3.29 (t, J = 7.7 Hz, 4H), 1.55-1.45 (m, 4H), 1.32 (h, J = 7.4 Hz, 4H), 0.91 (t, J = 7.3 Hz, 6H). ^{13}C NMR (DMSO 126 MHz): δ 223.39, 150.22, 148.74, 145.68, 133.88, 130.45, 129.03, 123.50, 123.24, 120.62, 120.41, 111.81, 50.30, 29.52, 20.13, 14.31. LCMS: 7 min method: RT: 3.08 min, M+H+: 309.50.

Synthesis of N,N-dioctyl-4-[(E)-2-(4-pyridyl)vinyl]aniline N2009-84.

To a mixture of 4-methylpyridine (0.22 mL, 2.25 mmol) and 4-(dioctylamino)benzaldehyde (0.86 g, 2.48 mmol) potassium tert-butoxide (0.38 g, 3.38 mmol) was added every hour for 4 h at 135 °C. After the last addition, the reaction mixture was stirred at 135 °C for further 1 h. Then, 4-methylpyridine (0.22 mL, 2.25 mmol) and potassium tert-butoxide (0.38 g, 3.38 mmol) were added and the reaction mixture was stirred at 135 °C for further 1 h. After cooling, water was added and the solution was concentrated. The residue was purified by flash column chromatography gradient elution of

petroleum ether / ethyl acetate (100 / 0 to 20 / 80) to give the desired as a yellow/orange oil (0.48 g, 51%). ^{1}H NMR (DMSO 500 MHz): δ 8.46-8.42 (m, 2H), 7.45-7.39 (m, 4H), 7.36 (d, J = 16.3 Hz, 1H), 6.86 (d, J = 16.3 Hz, 1H), 6.62 (d, J = 8.6 Hz, 2H), 3.27 (t, J = 7.9 Hz, 4H), 1.50 (p, J = 7.1 Hz, 4H), 1.32-1.20 (m, 20H), 0.89-0.81 (m, 6H). ^{13}C NMR (DMSO 126 MHz): δ 150.23, 148.73, 145.67, 133.86, 129.01, 123.25, 120.61, 120.41, 111.80, 50.56, 31.69, 29.32, 29.16, 27.32, 26.88, 22.53, 14.37. LCMS: 7 min method: RT: 8.65 min, M+H+: 421.61.

Scheme 3

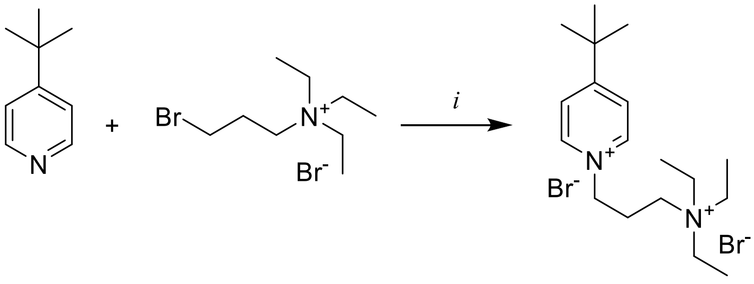

N2009-19-3 40%
UoS-13248

Reagents and conditions: *i*: anhydrous THF, sealed tube, 70 °C, 16 h.

Synthesis of 3-(4-tert-butylpyridin-1-ium-1-yl)propyl-triethyl-ammonium dibromide. **N2009-20-3; UoS-13248.**

A suspension of 3-bromopropyl(triethyl)ammonium bromide (0.35 g, 1.15 mmol) and 4-tert-butylpyridine (0.20 mL, 1.39 mmol) in anhydrous tetrahydrofuran (2 mL) was stirred in a sealed tube at 70 °C for 16 h. After cooling, the solvent was removed and the residue was purified by reverse phase HPLC eluting with water / acetonitrile = 95 / 5 to 0 / 100 in 30 min to give the desired compound as a colourless hygroscopic solid (0.20 g, 39%). ^{1}H NMR (MeOH 500 MHz): δ 9.19-9.15 (m, 2H), 8.25-8.20 (m, 2H), 4.92-4.83 (m, 2H), 3.59-3.51 (m, 2H), 3.47 (q, J = 7.3 Hz, 6H), 2.64-2.54 (m, 2H), 1.48 (s, 9H), 1.39 (t, J = 7.3 Hz, 9H).

Scheme 4

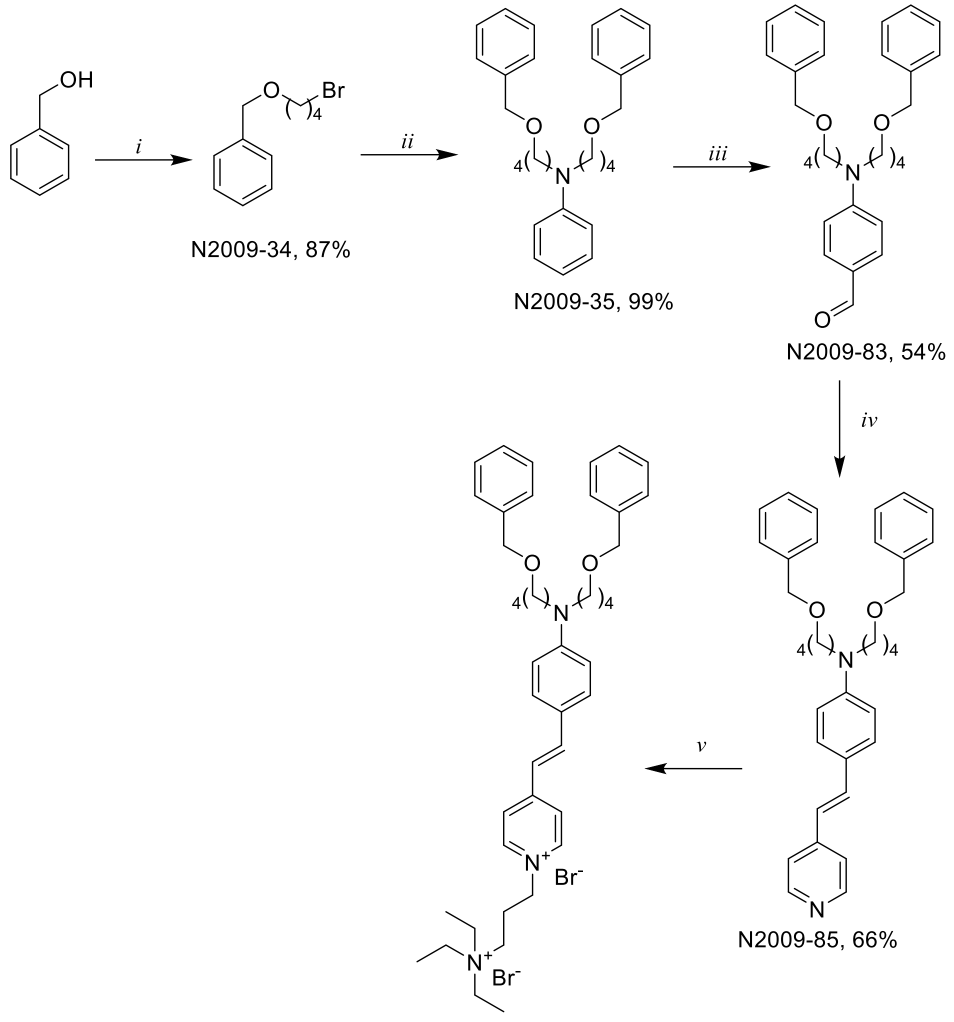

N2009-88-3, UoS13696, 15%

Reagents and conditions: *i*: 1,4-dibromobutane, sodium hydroxide, tetrabutylammonium bisulfate, water, 75 °C, 4 h; *ii*: aniline, potassium carbonate, 110 °C, 15 h; *iii*: $POCl_3$, DMF, 5 °C, 30 min, then 80 °C, 3.5 h; *iv*: 4-methylpyridine, potassium tert-butoxide, 135 °C, 5 h; *v*: 3-bromopropyl(triethyl)ammonium bromide, toluene, 120 °C, 40 h;

Synthesis of 4-bromobutoxymethylbenzene N2009-34.

A solution/suspension of benzyl alcohol (3.0 mL, 28.99 mmol), 1,4-dibromobutane (10.39 mL, 86.97 mmol), sodium hydroxide (3.48 g, 86.97 mmol) and tetrabutylammonium bisulfate (0.98 g, 2.90 mmol) in water (30 mL) was heated at 75 °C for 4 h. After cooling, water was added and the aqueous phase was extracted with dichloromethane. The organic phase was dried over $MgSO_4$, filtered and concentrated. The residue was purified by flash column chromatography gradient elution of petroleum ether / ethyl acetate (100 / 0 to 50 / 50) to give the desired compound (N2009-34-1) as a clear oil (6.10 g, 85%). ^{1}H NMR (DMSO 500 MHz): δ 7.38-7.24 (m, 5H), 4.45 (s, 2H), 3.53 (t, J = 6.7 Hz, 2H), 3.45 (t, J = 6.3 Hz, 2H), 1.87 (p, J = 6.9 Hz, 2H), 1.70-1.62 (m, 2H).

Synthesis of N,N-bis(4-benzyloxybutyl)aniline N2009-35.

A suspension of aniline (0.39 mL, 4.30 mmol), 4-bromobutoxymethylbenzene (3.13 g, 12.89 mmol) and potassium carbonate (2.37 g, 17.18 mmol) was stirred at 110 °C for 15 h. After cooling, water was added and the aqueous phase was extracted with DCM. The organic phase was dried over $MgSO_4$, filtered and concentrated. The residue was purified by flash column chromatography gradient elution of petroleum ether / ethyl acetate (100 / 0 to 80 / 20) to give the desired compound as a dark yellow oil (1.77 g, 99%). ^{1}H NMR (DMSO 500 MHz): δ 7.36-7.28 (m, 7H), 7.28-7.23 (m, 1H), 7.11-7.05 (m, 2H), 7.05-7.01 (m, 1H), 6.63-6.58 (m, 2H), 6.55-6.46 (m, 2H), 4.45 (s, 4H), 3.51-3.40 (m, 4H), 3.27-3.21 (m, 4H), 1.62-1.53 (m, 8H). ^{13}C NMR (DMSO 126 MHz): δ 170.75, 148.20, 139.14, 129.47, 129.26, 128.64, 127.86, 127.75, 115.28, 112.33, 111.99, 72.32, 72.28, 69.93, 60.19, 50.34, 43.07, 27.38, 27.09, 26.02, 24.05, 21.20. LCMS: 10 min method RT: 8.53 min, M+H+: 418.11.

Synthesis of 4-[bis(4-benzyloxybutyl)amino]benzaldehyde N2009-83.

To anhydrous N,N-dimethylformamide (10 mL) phosphorous oxychloride (0.40 mL, 4.31 mmol) was added dropwise at 0-5 °C and the reaction mixture was stirred at the same temperature for 30 min. Then, a solution of N,N-bis(4-benzyloxybutyl)aniline (1.80 g, 4.31 mmol) in anhydrous N,N-dimethylformamide (2 mL) was added dropwise and the reaction mixture was stirred at 80 °C for 3.5 h. After cooling, the reaction mixture was quenched with iced cold water and neutralised with 5N NaOH and extracted with ethyl acetate. The organic phase was washed with water (5 times), dried over $MgSO_4$, filtered and concentrated. The residue was purified by flash column chromatography gradient elution of petroleum ether / ethyl acetate (100 / 0 to 30 / 70) to give the desired compound (N2009-83-2) as a yellow oil (1.04 g, 54%). ^{1}H NMR (DMSO 500 MHz): δ 9.61 (s, 1H), 7.62-7.57 (m, 2H), 7.38-7.22 (m, 10H), 6.73 (d, J = 8.7 Hz, 2H), 4.45 (s, 4H), 3.46 (d, J = 5.9 Hz, 4H), 3.38 (d, J = 6.6 Hz, 4H), 1.63-1.54 (m, 8H). LCMS: 7 min method: RT=6.24 min, M+H+= 446.17.

Synthesis of N,N-bis(4-benzyloxybutyl)-4-[(E)-2-(4-pyridyl)vinyl]aniline N2009-85.

To a mixture of 4-methylpyridine (0.20 mL, 2.04 mmol) and 4-[bis(4-benzyloxybutyl)amino]benzaldehyde (1.0 g, 2.24 mmol) potassium tert-butoxide (0.34g, 3.06mmol) was added and the reaction mixture was stirred at at 135 °C. For 1 h. Then, potassium tert-butoxide (0.34g , 3.06mmol) was added at 135 °C every hour for 4 h. After the last addition, reaction mixture was stirred at 135 °C for further 1 h. After cooling, water was added and the solution was concentrated. The residue was purified by flash column chromatography gradient elution of petroleum ether / ethyl acetate (100 / 0 to 20 / 80) to give the desired compound as a dark yellow oil (0.70 g, 66%). ^{1}H NMR (DMSO 500 MHz): δ 8.48-8.42 (m, 2H), 7.46-7.42 (m, 2H), 7.41-7.24 (m, 13H), 6.85 (d, J = 16.3 Hz, 1H), 6.65 (d, J = 8.9 Hz, 2H), 4.45 (s, 4H), 3.46 (t, J = 5.4 Hz, 4H), 3.33-3.28 (m, 4H), 1.60-1.55 (m, 8H). ^{13}C NMR (DMSO 126 MHz): δ 150.25, 150.14, 148.66, 145.67, 139.13, 133.88, 129.00, 128.63, 127.88, 127.77, 120.63, 120.44, 111.88, 72.33, 69.90, 50.28, 27.01, 24.16. LCMS: 7 min method: RT: 3.78 min, $M+H^+$: 521.33.

Synthesis of 3-[4-[(E)-2-[4-[bis(4-benzyloxybutyl)amino]phenyl]vinyl]pyridin-1-ium-1-yl]propyl-triethyl-ammonium dibromide N2009-88-3 UoS-13696.

A solution of N,N-bis(4-benzyloxybutyl)-4-[(E)-2-(4-pyridyl)vinyl]aniline (0.30 g, 0.58 mmol) and 3-bromopropyl(triethyl)ammonium bromide (0.35 g, 1.15 mmol) in toluene (5 mL) was stirred at 120 °C for 40 h. After cooling, the solvent was removed under reduced pressure and the residue was purified by reverse phase HPLC eluting with water / acetonitrile = 95 / 5 to 0 / 100 in 20 min to give the desired

compound as a dark red sticky solid (0.13 g) slightly impure. A 60 mg portion was further purified by reverse phase HPLC eluting with water / acetonitrile = 95 / 5 to 0 / 100 in 20 min to give the desired compound as a dark red sticky solid (0.04 g, 8%). ^{1}H NMR (DMSO 500 MHz): δ 8.80 (d, J = 6.4 Hz, 2H), 8.09 (d, J = 6.5 Hz, 2H), 7.93 (d, J = 16.0 Hz, 1H), 7.53 (d, J = 8.5 Hz, 2H), 7.37-7.24 (m, 10H), 7.12 (d, J = 15.9 Hz, 1H), 6.73 (d, J = 8.7 Hz, 2H), 4.49 (t, J = 7.5 Hz, 2H), 4.45 (s, 4H), 3.47 (d, J = 5.8 Hz, 4H), 3.36 (d, J = 7.8 Hz, 4H), 3.27-3.19 (m, 8H), 2.30 (q, J = 8.1 Hz, 2H), 1.65-1.54 (m, 8H), 1.19 (t, J = 7.2 Hz, 9H). ^{13}C NMR (DMSO 126 MHz): δ 154.61, 150.48, 143.98, 143.06, 139.11, 131.06, 128.68, 127.80, 122.69, 112.06, 72.34, 69.86, 52.80, 26.93, 24.22, 23.59, 7.69. LCMS: 7 min method, RT: 4.95 min; compound does not ionised.

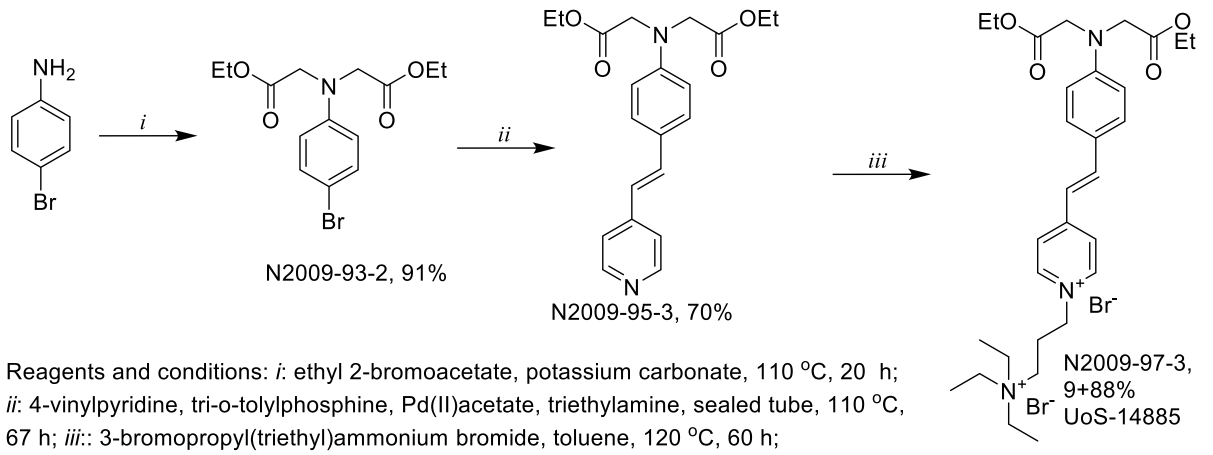

Reagents and conditions: *i*: ethyl 2-bromoacetate, potassium carbonate, 110 °C, 20 h; *ii*: 4-vinylpyridine, tri-o-tolylphosphine, Pd(II)acetate, triethylamine, sealed tube, 110 °C, 67 h; *iii*:: 3-bromopropyl(triethyl)ammonium bromide, toluene, 120 °C, 60 h;

Synthesis of ethyl 2-(4-bromo-N-(2-ethoxy-2-oxo-ethyl)anilino)acetate N2009-93-2.

A mixture of 4-bromoaniline (2.0 g, 11.63 mmol), ethyl 2-bromoacetate (5.16 mL, 46.51 mmol) and potassium carbonate (6.43 g, 46.51 mmol) were stirred at 110 °C for 20 h. After cooling, water was added and the aqueous phase was extracted with DCM. The organic phase was washed with brine, dried over $MgSO_4$, filtered and concentrated. The residue was purified by flash column chromatography gradient elution of petroleum ether / ethyl acetate (100 / 0 to 60 / 40) to give the desired compound as a yellow oil (3.65 g, 91%). ^{1}H NMR (DMSO 500 MHz): δ 7.31-7.26 (m, 2H), 6.54-6.48 (m, 2H), 4.18 (s, 4H), 4.11 (q, J = 7.1 Hz, 4H), 1.18 (t, J = 7.1 Hz, 6H). LCMS: 7 min method: RT: 4.61 min; M+H+: 343.94, 345.93.

Synthesis of ethyl 2-[N-(2-ethoxy-2-oxo-ethyl)-4-[(E)-2-(4-pyridyl)vinyl]anilino]acetate N2009-95-3.

A solution/suspension of ethyl 2-[4-bromo-N-(2-oxopentyl)anilino]acetate (2.0 g, 5.84 mmol), 4-vinylpyridine (0.79 mL, 7.31 mmol), tri-o-tolylphosphine (0.36 g, 1.17 mmol) and palladium (II) acetate (0.01 g, 0.06 mmol) in triethylamine (3 mL) was stirred in a sealed tube at 110 °C for 67 h. After cooling, the reaction mixture was diluted with DCM and the organic phase was washed with water, dried over $MgSO_4$, filtered and concentrated. The residue was purified by flash column chromatography gradient elution of petroleum ether / ethyl acetate (100 / 0 to 0 / 100) to give the desired compound as a yellow solid (1.51 g, 70%). ^{1}H NMR (DMSO 500 MHz): δ 8.50-8.45 (m, 2H), 7.50-7.44 (m, 4H), 7.40 (d, J = 16.4 Hz, 1H), 6.97 (d, J = 16.3 Hz, 1H), 6.63-6.57 (m, 2H), 4.24 (s, 4H), 4.13 (q, J = 7.1 Hz, 4H), 1.20 (t, J = 7.1 Hz, 6H). ^{13}C NMR (DMSO 126 MHz): δ 170.66, 150.30, 148.72, 145.39, 133.44, 128.70, 125.72, 122.08, 120.81, 112.53, 60.94, 53.09, 14.57. LCMS: 7 min method; RT: 0.90 min; M+H+: 369.22.

Synthesis of 3-[4-[(E)-2-[4-[bis(2-ethoxy-2-oxo-ethyl)amino]phenyl]vinyl]pyridin-1-ium-1-yl]propyl-triethyl-ammonium dibromide N2009-97-3 UoS-14885.

A solution of ethyl 2-[N-(2-ethoxy-2-oxo-ethyl)-4-[(E)-2-(4-pyridyl)vinyl]anilino]acetate (0.25 g, 0.68 mmol) and 3-bromopropyl(triethyl)ammonium bromide (0.41 g, 1.36 mmol) in anhydrous toluene (5 mL) was stirred in a sealed tube at 120 °C for 60 h. After cooling, the solvent was removed under reduced pressure and the residue was purified by reverse phase HPLC eluting with water / acetonitrile = 95 / 5 to 0 / 100 in 20 min to give the desired compound which was further purified by reverse phase HPLC eluting with water / acetonitrile = 95 / 5 to 0 / 100 in 20 min to give the desired compound as a pale red solid (0.04 g, 9%). ^{1}H NMR (DMSO 500 MHz): δ 8.87 (d, J = 6.5 Hz, 2H), 8.15 (d, J = 6.5 Hz, 2H), 7.96 (d, J = 16.1 Hz, 1H), 7.60 (d, J = 8.5 Hz, 2H), 7.25 (d, J = 16.0 Hz, 1H), 6.68 (d, J = 8.5 Hz, 2H), 4.52 (t, J = 7.5 Hz, 2H), 4.30 (s, 4H), 4.13 (q, J = 7.1 Hz, 4H), 3.27-3.24 (m, 8H), 2.31-2.28 (m, 2H), 1.22-1.17 (m, 15H). ^{13}C NMR (DMSO 126 MHz): δ 170.33, 150.52, 144.31, 130.48, 124.53, 123.22, 118.83, 112.76, 61.06, 52.97, 23.58, 14.57, 7.69. LCMS: 7 min method: RT: 1.11 min; M: 369.22

Scheme 5

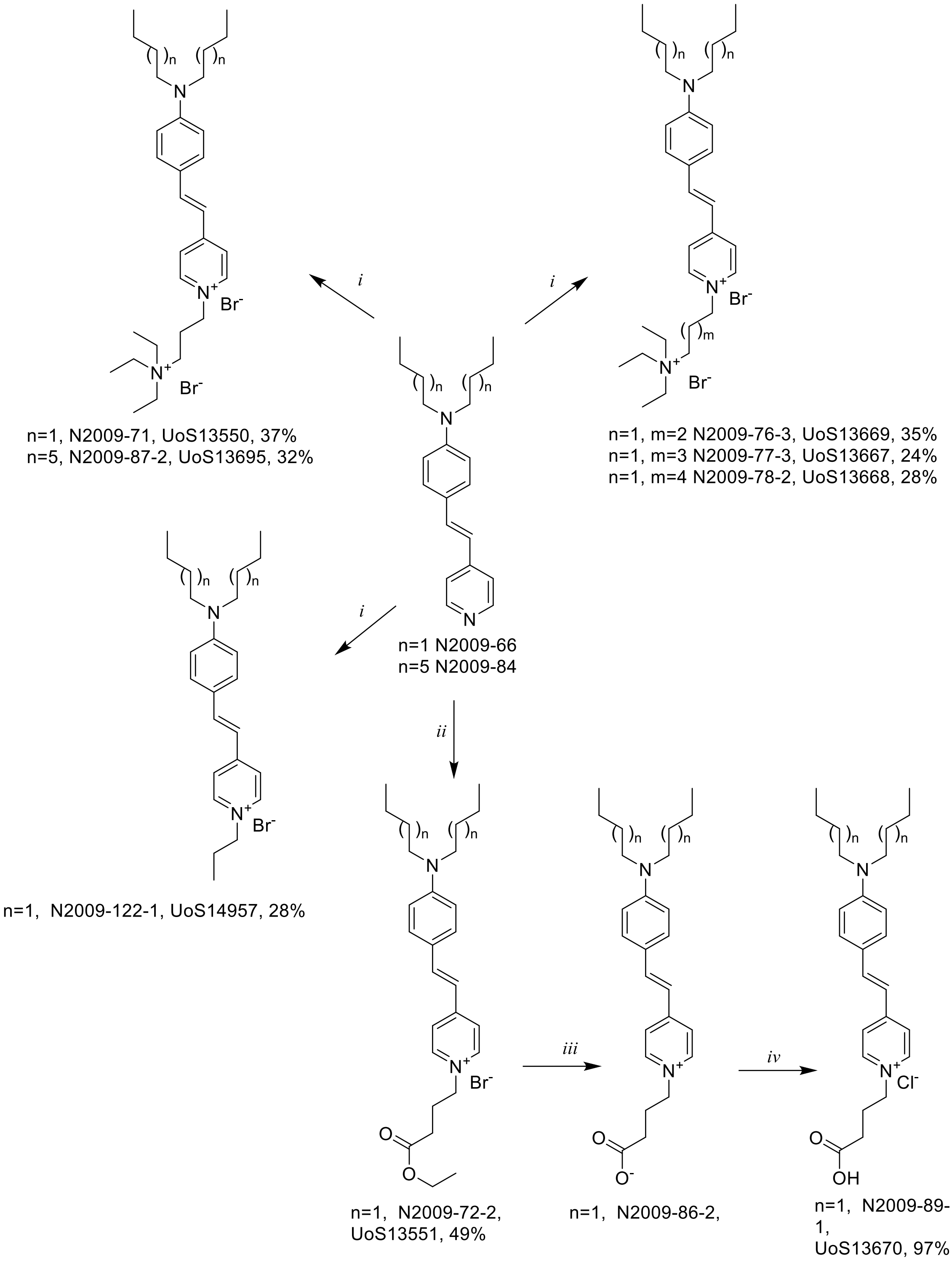

Reagents and conditions: *i*: appropriate 1-bromo-alkyl-triethylammonium bromide, anhydrous toluene, sealed tube 110-135 ^{o}C, 40-84 h; *ii*: ethyl 4-bromobutyrate, anhydrous toluene, sealed tube 110 ^{o}C, 60 h; *iii*: NaOH, water, room temperature, 6 h; *iv*: HCl, water, room temperature, 30 min.

Synthesis of 3-[4-[(E)-2-[4-(dibutylamino)phenyl]vinyl]pyridin-1-ium-1-yl]propyl-triethyl-ammonium dibromide FM1-43 – UoS-13550; N2009-71-1.

A solution of N,N-dibutyl-4-[(E)-2-(4-pyridyl)vinyl]aniline (0.15 g, 0.49 mmol) and 3-bromopropyl(triethyl)ammonium bromide (0.29 g, 0.97 mmol) in anhydrous toluene (8 mL) was stirred at 110 °C for 60 h and then at 135 °C for 24 h. After cooling, the solvent was removed under reduced pressure and the residue was purified by reverse phase HPLC eluting with water / acetonitrile = 95 / 5 to 0 / 100 in 20 min to give the desired compound as a dark red gum (0.11 g, 37%). ^{1}H NMR (DMSO 500 MHz): δ 8.80 (d, J = 6.4 Hz, 2H), 8.09 (d, J = 6.4 Hz, 2H), 7.93 (d, J = 16.0 Hz, 1H), 7.57 (d, J = 8.4 Hz, 2H), 7.13 (d, J = 15.9 Hz, 1H), 6.73 (d, J = 8.5 Hz, 2H), 4.49 (t, J = 7.5 Hz, 2H), 3.27-3.22 (m, 12H), 2.32-2.24 (m, 2H), 1.55-1.49 (m, 4H), 1.33 (h, J = 7.5 Hz, 4H), 1.18 (t, J = 7.1 Hz, 9H), 0.92 (t, J = 7.4 Hz, 6H). ^{13}C NMR (DMSO 126 MHz): δ 154.62, 150.53, 143.96, 131.10, 122.67, 112.00, 109.99, 52.81, 50.32, 29.52, 20.07, 14.30, 7.69. LCMS: 7 min method, RT: 0.70 min.

Synthesis of 3-[4-[(E)-2-[4-(dioctylamino)phenyl]vinyl]pyridin-1-ium-1-yl]propyl-triethyl-ammonium dibromide N2009-87-2 UoS-13695.

A solution of N,N-dioctyl-4-[(E)-2-(4-pyridyl)vinyl]aniline (0.20 g, 0.48 mmol) and 3-bromopropyl(triethyl)ammonium bromide (0.29 g, 0.95 mmol) in anhydrous toluene (5 mL) was stirred in a sealed tube for 40 h. After cooling, the solvent was removed under reduced pressure and the residue was purified by reverse phase HPLC eluting with water / acetonitrile = 95 / 5 to 0 / 100 in 20 min to give the desired compound as a dark red gum (0.11 g, 32%). ^{1}H NMR (MeOH 500 MHz): δ 8.76 (d, J = 6.7 Hz, 2H), 7.98 (d, J = 6.5 Hz, 2H), 7.83 (d, J = 15.9 Hz, 1H), 7.59 (d, J = 8.4 Hz, 2H), 7.05 (d, J = 15.9 Hz, 1H), 6.69 (d, J = 8.5 Hz, 2H), 4.63 (t, J = 7.4 Hz, 2H), 3.48-3.38 (m, 10H), 2.54-2.44 (m, 2H), 1.70-1.55 (m, 4H), 1.36-1.31 (m, 31H), 0.90 (t, J = 6.4 Hz, 6H). ^{13}C NMR (MeOH 126 MHz): δ 155.33, 150.69, 143.50, 143.07, 130.79, 122.31, 122.28, 115.81, 111.49, 109.98, 55.86, 53.18, 50.59, 31.58, 29.18, 29.04, 27.09, 26.68, 23.73, 22.33, 13.10, 6.66. LCMS: 7 min method, RT: 5.76 min.

Synthesis of 4-[4-[(E)-2-[4-(dibutylamino)phenyl]vinyl]pyridin-1-ium-1-yl]butyl-triethyl-ammonium dibromide N2009-76-3 UoS-13669.

A solution of N,N-dibutyl-4-[(E)-2-(4-pyridyl)vinyl]aniline (0.10 g, 0.32 mmol) and 4-bromobutyl(triethyl)ammonium bromide (0.21 g, 0.65 mmol) in anhydrous toluene (5 mL) was stirred in a sealed tube at 130 °C for 60 h. After cooling, the solvent was removed under reduced pressure and the residue was purified by two subsequent reverse phase HPLC eluting with water / acetonitrile = 95 / 5 to 0 / 100 in 20 min to give the desired compound as a red gum (0.07 g, 34%). ^{1}H NMR (DMSO 500 MHz): δ 8.74 (d, J = 6.5 Hz, 2H), 8.06 (d, J = 6.5 Hz, 2H), 7.91 (d, J = 16.0 Hz, 1H), 7.56 (d, J = 8.5 Hz, 2H), 7.13 (d, J = 16.0 Hz, 1H), 6.73 (d, J = 8.5 Hz, 2H), 4.45 (t, J = 7.3 Hz, 2H), 3.35 (t, J = 7.7 Hz, 4H), 3.25-3.21 (m, 6H), 3.17 (t, J = 8.5 Hz, 2H), 1.92 (p, J = 7.6 Hz, 2H), 1.63 (p, J = 8.4 Hz, 2H), 1.55-1.49 (m, 4H), 1.33 (h, J = 7.4 Hz, 4H), 1.17 (t, J = 7.1 Hz, 9H), 0.92 (t, J = 7.4 Hz, 6H). ^{13}C NMR (DMSO 126 MHz): δ 179.26, 172.98, 143.82, 131.03, 122.74, 111.99, 52.62, 50.31, 29.52, 20.07, 14.30, 7.66. LCMS: 7 min method: RT: 1.29 min.

Synthesis of 5-[4-[(E)-2-[4-(dibutylamino)phenyl]vinyl]pyridin-1-ium-1-yl]pentyl-triethyl-ammonium dibromide N2009-77-3 UoS-13667.

A solution of N,N-dibutyl-4-[(E)-2-(4-pyridyl)vinyl]aniline (0.10 g, 0.32 mmol) and 5-bromopentyl(triethyl)ammonium bromide (0.21 g, 0.65 mmol) in anhydrous toluene (5 mL) was stirred in a sealed tube at 130 °C for 60 h. After cooling, the solvent was removed under reduced pressure and the residue was purified by reverse phase HPLC eluting with water / acetonitrile = 95 / 5

to 0 / 100 in 20 min to give the desired compound as a red gum (0.05 g, 24%). ^{1}H NMR (MeOH 500 MHz): δ 8.72-8.63 (m, 2H), 8.05-7.96 (m, 2H), 7.85 (d, J = 15.9 Hz, 1H), 7.70-7.57 (m, 2H), 7.09 (d, J = 16.1 Hz, 1H), 6.86-6.68 (m, 2H), 4.52 (t, J = 7.5 Hz, 2H), 3.46-3.39 (m, 4H), 3.36 (q, J = 7.3 Hz, 6H), 3.29-3.22 (m, 2H), 2.11 (p, J = 7.6 Hz, 2H), 1.85-1.79 (m, 2H), 1.60 (p, J = 7.7 Hz, 4H), 1.50 (p, J = 7.7 Hz, 2H), 1.40 (h, J = 7.5 Hz, 4H), 1.34-1.27 (m, 9H), 0.98 (t, J = 7.4 Hz, 6H). ^{13}C NMR (MeOH 126 MHz): δ 143.01, 130.55, 122.39, 109.99, 59.31, 56.43, 52.70, 30.28, 22.67, 20.99, 19.77, 12.89, 6.47. LCMS: 7 min method, RT: 0.70 min.

Synthesis of 6-[4-[(E)-2-[4-(dibutylamino)phenyl]vinyl]pyridin-1-ium-1-yl]hexyl-triethyl-ammonium dibromide N2009-78-2 UoS-13668.

A solution of N,N-dibutyl-4-[(E)-2-(4-pyridyl)vinyl]aniline (0.10 g, 0.32 mmol) and 6-bromohexyl(triethyl)ammonium bromide (0.22 g, 0.65 mmol) in anhydrous toluene (5 mL) was stirred in a sealed tube at 130 °C for 60 h. After cooling, the solvent was removed under reduced pressure and the residue was purified by reverse phase HPLC eluting with water / acetonitrile = 95 / 5 to 0 / 100 in 20 min to give the desired compound as a red gum (0.06 g, 28%). ^{1}H NMR (MeOH 500 MHz): δ 8.65 (d, J = 6.5 Hz, 2H), 7.98 (d, J = 6.5 Hz, 2H), 7.83 (d, J = 16.0 Hz, 1H), 7.60 (d, J = 8.5 Hz, 2H), 7.07 (d, J = 15.9 Hz, 1H), 6.73 (d, J = 8.5 Hz, 2H), 4.47 (t, J = 7.5 Hz, 2H), 3.42-3.34 (m, 10H), 3.27-3.18 (m, 2H), 2.07-1.99 (m, 2H), 1.79-1.69 (m, 2H), 1.60 (p, J = 7.7 Hz, 4H), 1.55-1.45 (m, 4H), 1.39 (h, J = 7.5 Hz, 4H), 1.30 (t, J = 6.8 Hz, 9H), 0.98 (t, J = 7.4 Hz, 6H). ^{13}C NMR (MeOH 126 MHz): δ 154.90, 142.93, 130.60, 122.28, 115.95, 111.60, 59.52, 56.69, 52.62, 50.42, 30.53, 29.19, 25.26, 21.18, 19.82, 12.95, 6.50. LCMS: 7 min method: RT: 0.51 min.

Synthesis of N,N-dibutyl-4-[(E)-2-(1-propylpyridin-1-ium-4-yl)vinyl]aniline bromideN2009-122-1 UoS-14957.

A solution of N,N-dibutyl-4-[(E)-2-(4-pyridyl)vinyl]aniline (0.10 g, 0.41 mmol) and 1-bromopropane (0.08 mL, 0.83 mmol) in anhydrous toluene (3 mL) was stirred in a sealed tube at 120 ° for 60 h. After cooling, the solvent was removed under reduced pressure and the residue was purified by reverse phase HPLC eluting with water / acetonitrile = 95 / 5 to 0 / 100 in 20 min to give the desired compound as a red gum (0.05 g, 28%). ^{1}H NMR (DMSO 500 MHz): δ 8.74 (d, J = 6.4 Hz, 2H), 8.04 (d, J = 6.4 Hz, 2H), 7.90 (d, J = 16.0 Hz, 1H), 7.55 (d, J = 8.5 Hz, 2H), 7.11 (d, J = 16.0 Hz, 1H), 6.72 (d, J = 8.5 Hz, 2H), 4.37 (t, J = 7.2 Hz, 2H), 3.35 (t, J = 7.7 Hz, 4H), 1.96-1.84 (m, 2H), 1.52 (p, J = 7.9 Hz, 4H), 1.33 (h, J = 7.4 Hz, 4H), 0.92 (t, J = 7.4 Hz, 6H), 0.88 (t, J = 7.3 Hz, 3H). ^{13}C NMR (DMSO 500 MHz): δ 154.23, 150.36, 143.83, 142.68, 130.98, 122.65, 122.15, 116.91, 111.93, 60.84, 50.30, 29.50, 24.36, 20.07, 14.31, 10.72. LCMS: 7 min method: RT: 3.12 min; M-Br-: 351.33.

Synthesis of ethyl 4-[4-[(E)-2-[4-(dibutylamino)phenyl]vinyl]pyridin-1-ium-1-yl]butanoate bromide N2009-72-2 UoS-13551.

A solution of N,N-dibutyl-4-[(E)-2-(4-pyridyl)vinyl]aniline (0.15 g, 0.49 mmol) and ethyl 4-bromobutyrate (0.14 mL, 0.97 mmol) in anhydrous toluene (7 mL) was stirred at 110 °C for 60 h. After cooling, the solvent was removed under reduced pressure and the residue was purified by reverse phase HPLC eluting with water / acetonitrile = 95 / 5 to 0 / 100 in 20 min to give the desired compound as a dark red gum (0.12 g, 49%). ^{1}H NMR (MeOH 500 MHz): δ 8.56 (d, J = 6.5 Hz, 2H), 7.94 (d, J = 6.5 Hz, 2H), 7.79 (d, J = 16.0 Hz, 1H), 7.58 (d, J = 8.5 Hz, 2H), 7.01 (d, J = 15.9 Hz, 1H), 6.69 (d, J = 8.8 Hz, 2H), 4.44 (t, J = 7.4 Hz, 2H), 4.11 (q, J = 7.1 Hz, 2H), 3.41-3.29 (m, 4H), 2.47 (t, J = 7.2 Hz, 2H), 2.22 (p, J

= 7.5 Hz, 2H), 1.63-1.52 (m, 4H), 1.38 (h, J = 7.5 Hz, 4H), 1.24 (t, J = 7.1 Hz, 3H), 0.97 (t, J = 7.4 Hz, 6H). ^{13}C NMR (MeOH 126 MHz): δ 172.31, 154.98, 150.60, 143.11, 142.87, 122.31, 122.04, 115.86, 111.47, 60.50, 58.78, 50.32, 30.00, 29.26, 26.02, 19.85, 13.20, 13.01. LCMS: 7 min method: RT=3.21 min, M-Br^-= 423.34.

Synthesis of 4-[4-[(E)-2-[4-(dibutylamino)phenyl]vinyl]pyridin-1-ium-1-yl]butanoate N2009-86-2.

To a solution of ethyl 4-[4-[(E)-2-[4-(dibutylamino)phenyl]vinyl]pyridin-1-ium-1-yl]butanoate bromide (0.17 g, 0.34 mmol) in water (20 mL) sodium hydroxide (0.02 g, 0.51 mmol) was added and the reaction mixture was stirred at room temperature for 4 h. Then, sodium hydroxide (0.01 g, 0.25 mmol) was added and the stirring was continued for further 2 h. Then, the solvent was removed under reduced pressure and the residue was purified by reverse phase HPLC eluting with water / acetonitrile = 95 / 5 to 0 / 100 in 20 min to give the desired compound as a red solid (0.10 g, 75%). ^{1}H NMR (DMSO 500 MHz): δ 8.77 (d, J = 6.5 Hz, 2H), 8.00 (d, J = 6.5 Hz, 2H), 7.86 (d, J = 16.0 Hz, 1H), 7.55 (d, J = 8.5 Hz, 2H), 7.09 (d, J = 16.0 Hz, 1H), 6.71 (d, J = 8.6 Hz, 2H), 4.43 (t, J = 7.0 Hz, 2H), 3.38-3.32 (m, 4H), 1.96 (p, J = 7.0 Hz, 2H), 1.78 (t, J = 6.8 Hz, 2H), 1.52 (p, J = 7.6 Hz, 4H), 1.33 (h, J = 7.4 Hz, 4H), 0.92 (t, J = 7.4 Hz, 6H). ^{13}C NMR (DMSO 126 MHz): δ 173.07, 153.93, 150.31, 144.13, 142.34, 130.88, 122.54, 122.25, 117.09, 111.94, 59.96, 50.31, 35.22, 29.52, 28.95, 20.08, 14.30. LCMS: 7 min method; RT: 2.72 min, M+H^+= 395.26.

Synthesis of 4-[4-[(E)-2-[4-(dibutylamino)phenyl]vinyl]pyridin-1-ium-1-yl]butanoic acid chloride N2009-89-1 UoS-13670.

A mixture of 4-[4-[(E)-2-[4-(dibutylamino)phenyl]vinyl]pyridin-1-ium-1-yl]butanoate (85.0 mg, 0.22 mmol) was suspended in water (5 mL) and 1M HCl (0.22 mL, 0.22 mmol) was added and the reaction mixture was stirred at room temperature for 30 min. Then, the water was removed under reduced pressure to give the desired compound as a pale red solid (90 mg, 97%). ^{1}H NMR (DMSO 500 MHz): δ 8.76 (d, J = 6.9 Hz, 2H), 8.11-7.98 (m, 2H), 7.91 (d, J = 16.0 Hz, 1H), 7.63-7.52 (m, 2H), 7.14 (d, J = 15.8 Hz, 1H), 6.83-6.64 (m, 2H), 4.44 (t, J = 7.2 Hz, 2H), 3.36 (t, J = 7.5 Hz, 4H), 2.33 (t, J = 7.4 Hz, 2H), 2.21-2.02 (m, 2H), 1.60-1.44 (m, 4H), 1.32 (h, J = 7.5 Hz, 4H), 0.91 (t, J = 7.4 Hz, 6H). LCMS: 7 min method: RT: 2.83 min, M-Cl^-: 395.43.

Scheme 6

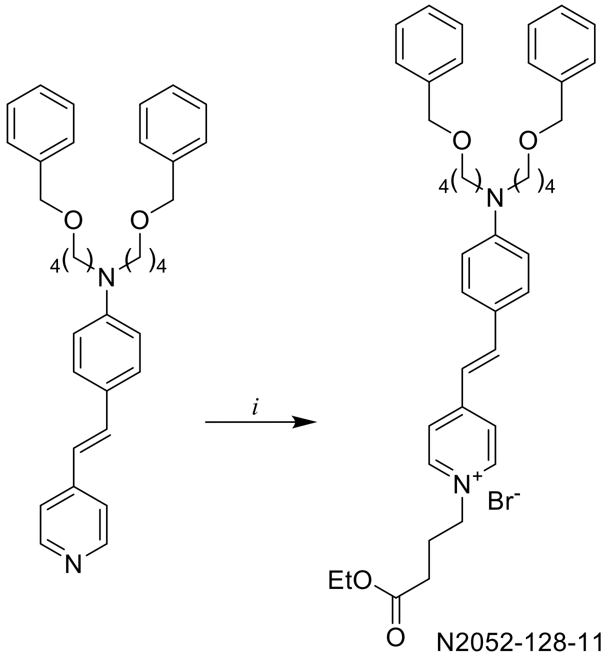

N2052-128-11, UoS28552, 11%

Reagents and conditions: *i*: ethyl 4-bromobutyrate, toluene, 130 ^{o}C, microwave, 3 x 1 h

Synthesis of ethyl 4-[4-[(E)-2-[4-[bis(4-benzyloxybutyl)amino]phenyl]vinyl]pyridin-1-ium-1-yl]butanoate bromide N2052-128-11 UoS-28552.

A solution of N,N-bis(4-benzyloxybutyl)-4-[(E)-2-(4-pyridyl)vinyl]aniline (0.15 g, 0.29 mmol) and ethyl 4-bromobutyrate (0.05 mL, 0.37 mmol) in toluene (1.5 mL) was stirred in a sealed tube in a microwave at 130 °C for 1 h. Then, ethyl 4-bromobutyrate (0.11 mL, 0.75 mmol) was added and the reaction mixture was stirred in a sealed tube in a microwave at 130 °C for 1.5 h. Then, ethyl 4-bromobutyrate (0.11 mL, 0.75 mmol) was added and the reaction mixture was stirred in a microwave at 130 °C for 1 h. After cooling, the solvent was removed under reduced pressure and the residue was purified by reverse phase HPLC eluting with water / acetonitrile = 95 / 5 to 0 / 100 in 20 min to give the desired compound as a red gum (24 mg, 11%) ^{1}H NMR (DMSO 500 MHz): δ 8.72 (d, J = 6.6 Hz, 2H), 8.04 (d, J = 6.6 Hz, 2H), 7.89 (d, J = 16.0 Hz, 1H), 7.54-7.49 (m, 2H), 7.37-7.24 (m, 10H), 7.09 (d, J = 16.0 Hz, 1H), 6.73 (d, J = 8.8 Hz, 2H), 4.45 (s,4H), 4.43 (t, J = 7.2 Hz, 2H),4.02 (q, J = 7.1 Hz, 2H), 3.52-3.42 (m, 4H), 3.40-3.33 (m, 4H), 2.40 (t, J = 7.4 Hz, 2H), 2.15 (p, J = 7.3 Hz, 2H), 1.64-1.53 (m, 8H), 1.16 (t, J = 7.1 Hz, 3H). LCMS: 4 min method: RT: 2.66 min; M+H^{+} (-Br^{-}): 635.41.

Scheme 7

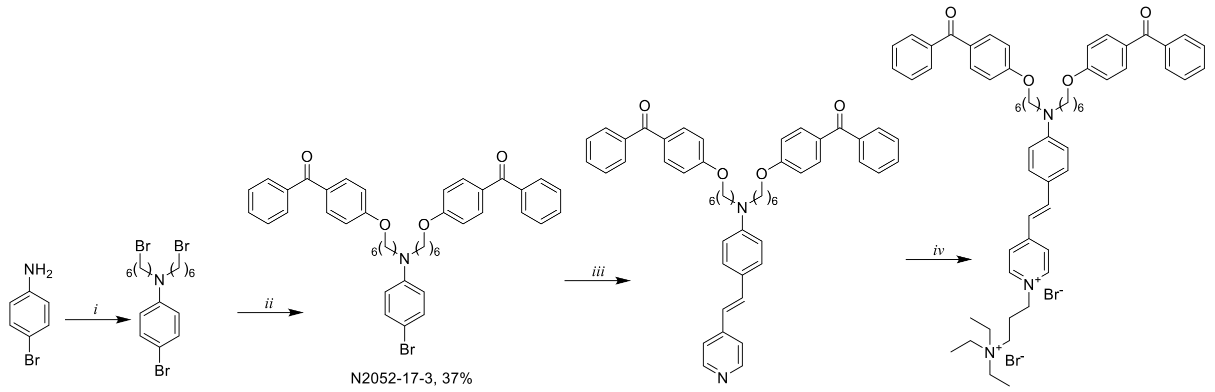

N2052-7-2, 38% N2052-17-3, 37% N2052-21-3, 39% N2052-27-3, UoS16327, 36%

Reagents and conditions: *i*: 1,6-dibromohexane, pptassium carbonate, 120 °C, 18 h; *ii*: (4-hydroxyphenyl)-phenyl-methanone, potassium carbonate, DMF, room temperature, 18 h; *iii*: 4-vinylpyridine, tri-o-tolylphosphine, Pd(II)acetate, triethylamine, sealed tube, 110 °C, 48 h; *iv*:3-bromopropyl(triethyl)ammonium bromide, toluene, 110 °C, 65 h

Synthesis of [4-bromo-N,N-bis(6-bromohexyl)aniline N2052-7-2.

A suspension of 4-bromoaniline (1.47 mL, 8.72 mmol), 1,6-dibromohexane (5.37 mL, 34.88 mmol) and potassium carbonate (4.82 g, 34.88 mmol) was heated at 120 °C for 18 h. After cooling, water was added and the aqueous phase was extracted with DCM. The organic phase was washed with brine, dried over $MgSO_4$, filtered and concentrated. The residue was purified by flash column chromatography eluting with petroleum ether, then gradient elution of petroleum ether / ethyl acetate (100 / 0 to 50 / 50) to give the desired compound impure which was further purified by flash column chromatography eluting with petroleum ether, then gradient elution of petroleum ether / ethyl acetate (100 / 0 to 60 / 40) to give the desired compound as a yellow oil (1.67 g, 38%). ^{1}H NMR (DMSO 500 MHz): δ 7.25-7.19 (m, 2H), 6.62-6.52 (m, 2H), 3.53-3.48 (m, 4H), 3.24-3.17 (m, 4H), 1.82-1.75 (m, 4H), 1.52-1.35 (m, 8H), 1.34-1.25 (m, 4H).

Synthesis of [4-[6-[N-[6-(4-benzoylphenoxy)hexyl]-4-bromo-anilino]hexoxy]phenyl]-phenyl-methanone N2052-17-3.

To a solution of 4-bromo-N,N-bis(6-bromohexyl)aniline (3.61 mL, 3.31 mmol) and anhydrous potassium carbonate (0.92 g, 6.62 mmol) in anhydrous N,N-dimethylformamide (20 mL) a solution of (4-hydroxyphenyl)-phenyl-methanone (0.66 g, 3.31 mmol) in anhydrous N,N-dimethylformamide (5 mL) was added dropwise and the reaction mixture was stirred at room temperature for 18 h. After this period, the solvent was concentrated and the residue was dissolved in ethyl acetate and water. The aqueous phase was extracted with ethyl acetate and the combined organic phase was washed with saturated solution of $NaHCO_3$ (5 times), water (once), dried over $MgSO_4$, filtered and concentrated. The residue was purified by flash column chromatography eluting with petroleum ether, then gradient elution of petroleum ether / ethyl acetate (100 / 0 to 0 / 100) to give the desired compound bis-benzophenone derivative as a yellow oil (0.89 g, 37%). The mono-benzophenone derivative was also isolated as a yellow oil (0.59 g, 29%). ^{1}H NMR (DMSO 500 MHz): δ 7.74-7.69 (m, 4H), 7.69-7.64 (m, 4H), 7.64-7.60 (m, 2H), 7.56-7.50 (m, 4H), 7.25-7.17 (m, 2H), 7.08-7.02 (m, 4H), 6.60-6.51 (m, 2H), 4.06 (t, J = 6.4 Hz, 4H), 3.26-3.17 (m, 4H), 1.80-1.68 (m, 4H), 1.58-1.39 (m, 8H), 1.40-1.24 (m, 4H).

Synthesis of [4-[6-[N-[6-(4-benzoylphenoxy)hexyl]-4-[(E)-2-(4-pyridyl)vinyl]anilino]hexoxy]phenyl]-phenyl-methanone N2052-21-3.

A solution/suspension of [4-[6-[N-[6-(4-benzoylphenoxy)hexyl]-4-bromo-anilino]hexoxy]phenyl]-phenyl-methanone (0.88 g, 1.20 mmol), 4-vinylpyridine (0.16 mL, 1.50 mmol), tri-o-tolylphosphine (0.07 g, 0.24 mmol) and palladium (II) acetate (0.01 g, 0.040 mmol) in triethylamine (3 mL) (compound not completely soluble) was stirred in a sealed tube at 110 °C for 48 h. After cooling, the reaction mixture was diluted with DCM and the organic phase was washed with water, dried over $MgSO_4$, filtered and concentrated. The residue was purified by flash column chromatography gradient elution of petroleum ether / ethyl acetate (100 / 0 to 0 / 100) to give the desired compound as an orange solid (0.35 g, 39%). ^{1}H NMR (DMSO 500 MHz): δ 8.48-8.39 (m, 2H), 7.74-7.70 (m, 4H), 7.69-7.60 (m, 6H), 7.53 (t, J = 7.7 Hz, 4H), 7.47-7.39 (m, 4H), 7.36 (d, J = 16.3 Hz, 1H), 7.07-7.04 (m, 4H), 6.86 (d, J = 16.3 Hz, 1H), 6.65 (d, J = 8.7 Hz, 2H), 4.07 (t, J = 6.5 Hz, 4H), 3.30-3.28 (m, 4H), 1.75 (p, J = 6.8 Hz, 4H), 1.55 (p, J = 7.8 Hz, 4H), 1.52-1.42 (m, 4H), 1.42-1.32 (m, 4H). LCMS: 7 min method: RT, 5.23 min; $M+H^+$: 757.64.

Synthesis of 3-[4-[(E)-2-[4-[bis[6-(4-benzoylphenoxy)hexyl]amino]phenyl]vinyl]pyridin-1-ium-1-yl]propyl-triethyl-ammonium dibromide N2052-27-3 UoS-16327.

A solution/suspensio of [4-[6-[N-[6-(4-benzoylphenoxy)hexyl]-4-[(E)-2-(4-pyridyl)vinyl]anilino]hexoxy]phenyl]-phenyl-methanone (0.10 g, 0.13 mmol) and 3-bromopropyl(triethyl)ammonium bromide (0.16 mL, 0.26 mmol) in toluene (3mL) was heated at 110 °C in a sealed tube for 65 h. After cooling, the solvent was removed and the residue was purified by reverse phase HPLC eluting with water / acetonitrile = 95 / 5 to 0 / 100 in 20 min to give the desired compound impure which was further purified by reverse phase HPLC eluting with water / acetonitrile = 95 / 5 to 0 / 100 in 20 min to give the desired compound as a red solid (0.05 g, 36%). ^{1}H NMR (DMSO 500 MHz): δ 8.77 (d, J = 6.5 Hz, 2H), 8.07 (d, J = 6.4 Hz, 2H), 7.92 (d, J = 16.0 Hz, 1H), 7.72 (d, J = 8.7 Hz, 4H), 7.71-7.61 (m, 6H), 7.54 (q, J = 8.0, 7.5 Hz, 6H), 7.12 (d, J = 15.9 Hz, 1H), 7.06 (d, J = 8.4 Hz, 4H), 6.75 (d, J = 8.6 Hz, 2H), 4.47 (t, J = 7.5 Hz, 2H), 4.08 (t, J = 6.3 Hz, 4H), 3.39-3.34 (m, 4H), 3.29-3.22 (m, 8H), 2.28 (t, J = 8.1 Hz, 2H), 1.76 (p, J = 6.9 Hz, 4H), 1.58 (p, J = 7.8 Hz, 4H), 1.48 (p, J = 7.3 Hz, 4H), 1.43-1.35 (m, 4H), 1.18 (t, J = 7.1 Hz, 9H). ^{13}C NMR (DMSO 126 MHz): δ 194.83, 162.87, 143.95, 138.27, 132.62, 132.50, 129.63, 122.67, 114.78, 68.30, 52.82, 28.94, 27.28, 26.50, 25.79, 7.67.

Synthesis of N,N-dibutyl-4-[(E)-2-[1-[3-[4-[(E)-2-[4-(dibutylamino)phenyl]vinyl]pyridin-1-ium-1-yl]propyl]pyridin-1-ium-4-yl]vinyl]aniline dibromide N2009-98-2 UoS-13698.

A solution of N,N-dibutyl-4-[(E)-2-(4-pyridyl)vinyl]aniline (0.1g, 0.32 mmol) and 1,3-dibromopropane (0.02mL, 0.16 mmol) in anhydrous toluene (4 mL) was stirred under a nitrogen atmosphere in a sealed tube at 120 °C for 30 h. After cooling, the solvent was removed under reduced pressure and the residue was purified by reverse phase HPLC eluting with water / acetonitrile = 95 / 5 to 0 / 100 in 20 min to give the desired compound as a dark red sticky solid (0.065 g, 24%). ^{1}H NMR (DMSO 500 MHz): δ 8.75 (d, J = 6.6 Hz, 4H), 8.06 (d, J = 6.5 Hz, 4H), 7.92 (d, J = 16.0 Hz, 2H), 7.55 (d, J = 8.6 Hz, 4H), 7.13 (d, J = 16.0 Hz, 2H), 6.72 (d, J = 8.6 Hz, 4H), 4.53 (t, J = 7.2 Hz, 4H), 3.35 (t, J = 7.6 Hz, 8H), 2.61 – 2.54 (m, 2H), 1.52 (p, J = 7.4 Hz, 8H), 1.33 (h, J = 7.4 Hz, 8H), 0.92 (t, J = 7.4 Hz, 12H). ^{13}C NMR (DMSO 126 MHz): δ 154.56, 150.50, 143.92, 143.03, 131.08, 122.74, 122.17, 116.89, 111.98, 56.42, 50.31, 31.75, 29.53, 20.07, 14.30. LCMS: 7 min method: RT: 3.16 min; $M-2Br^-$: 657.15.
