# Supplementary material for: Charge and lipophilicity are required for effective block of the hair-cell mechano-electrical transducer channel by FM1-43 and its derivatives
Source: Front Cell Dev Biol. 2023 Oct 10;11:1247324. doi: 10.3389/fcell.2023.1247324 (PMC10601989; doi:10.3389/fcell.2023.1247324)
Supplement: Supplementary file 2 [file DataSheet1.pdf]

Supplemental Methods, Results and Figures

Supplemental Methods

Data analysis for the zebrafish dye loading assay

Mean intensity data for each sample were background corrected to adjacent non-neuromast cells towards the head of the larvae. All data were then shifted by the minimum data value to deal with a small number of slightly negative values resulting from the background correction. A gamma-distributed generalized linear mixed-effects model (GLMM) was used to compare intensity responses for each compound against the parent compound, 13550. Intensity data + 1 were used as the response variable to avoid creation of log(0) in the computation. The identity of the compound, natural logarithm-transformed concentration, and natural logarithm-transformed treatment time were fitted as fully interacting fixed effects. The three-way interaction was removed, based on a likelihood ratio test, adjusted for small sample size using $\hat{c}$ of the more complex model. Two-way interactions were retained and compared against the parent compound, 13550. Replicates were modelled as random intercepts. All statistical analyses were conducted using R, version 4.1.2 (R Core Team, 2019). Additional R packages were: glmmTMB (Brooks et al., 2017) for mixed-effects models, AICcmodavg (Mazerolle, 2020) for model selection, and ggplot2 (Wickham, 2016) for data visualisation.

Data analysis for the mouse dye loading assay

The same mixed-effects model was fitted to fluorescence intensity mouse data, based on the identity of the compound and natural logarithm-transformed time as interacting fixed effects, since only one concentration was assessed. Unlike the fluorescence intensity analysis in zebrafish, since individual cochlear cultures were followed through time, longitudinal data were accounted for by inclusion of first order autoregressive correlation between time points grouped by replicate.

Supplemental Results

Analysis of loading properties of FM1-43 and its derivatives in zebrafish lateral line hair cells as a function of time and concentration and mouse cochlear cultures as a continuous time series

The ability of 13550 and its derivatives to load into the hair cells was assessed as a function of both time and concentration for zebrafish lateral line hair cells, and as a function of time for mouse cochlear culture hair cells at the single concentration that was used in this study (i.e., 0.3 µM) (Supplementary Figure S1, supported by Supplementary Table S1, zebrafish, and Supplementary Table S2, mouse). The fluorescence of compound 13550, like commercially available FM1-43, shows a strong dependence on both time and concentration in zebrafish, and on time in mouse hair cells. By comparison, compounds 14885, 13670, and 13698, that either failed to load or showed a significant reduction in fluorescence compared to 13550, displayed similar loading behaviour in both mouse OHCs and zebrafish neuromasts. Compounds 13957 and 13668, both of which showed a significant increase in fluorescence compared to 13550, also load in a similar manner in mouse and zebrafish. Compounds 16327 and 13667 behaved in a similar manner to 13550 in mouse OHCs, but they showed a reduction in fluorescence in zebrafish. Compounds 13551, 13669, and 28552 showed an increase in mouse, compared to 13550, but a decrease in zebrafish. The remaining compounds are in broad alignment regarding loading compared to the parent compounds, although this is time and concentration dependent. None of the compounds showed a significantly faster rate of uptake than 13550, though 14957 did show a stronger response to increasing concentration.

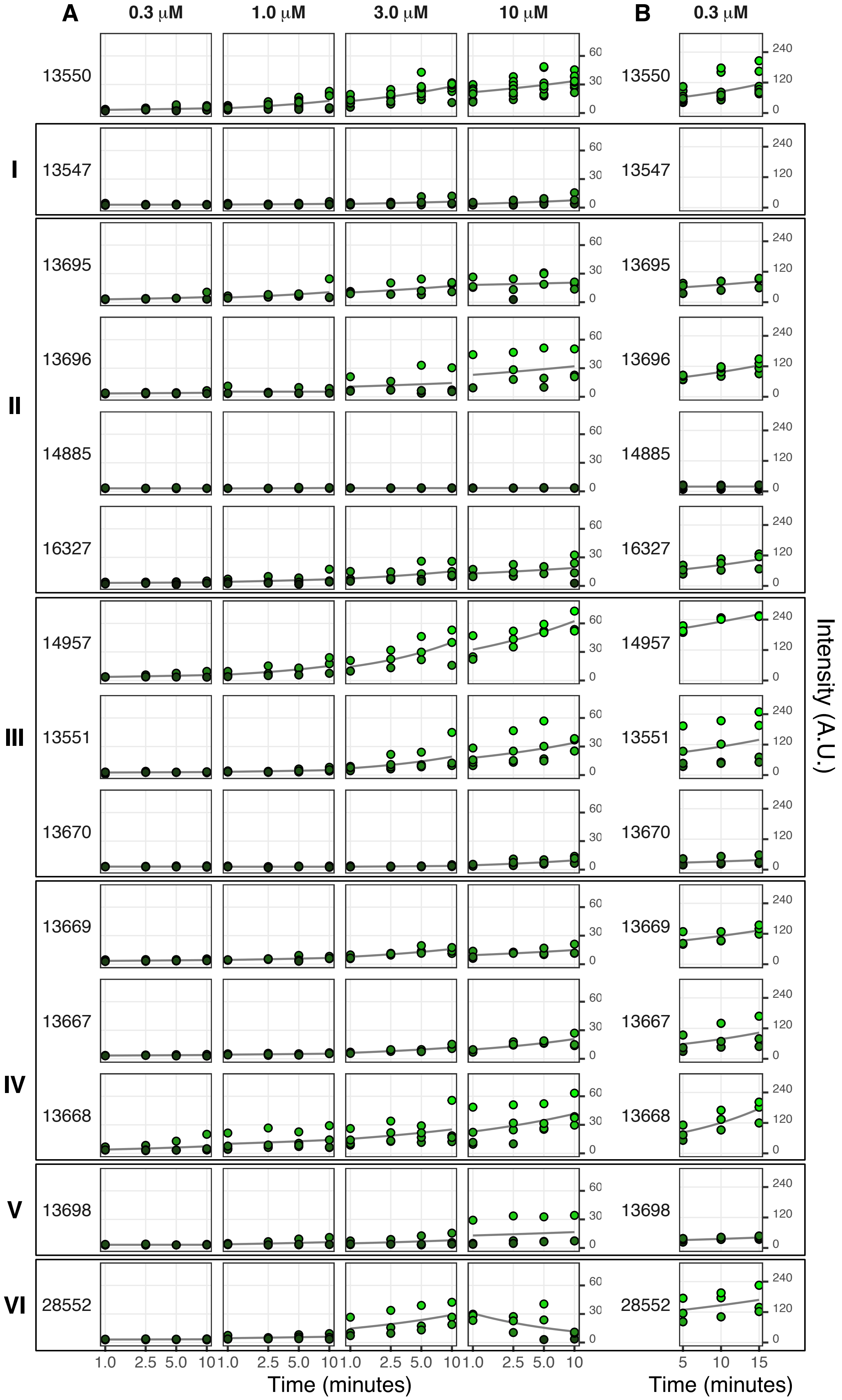
A
0.3 μM
1.0 μM
3.0 μM
10 μM
B
0.3 μM
I
II
III
IV
V
VI
13550
13547
13695
13696
14885
16327
14957
13551
13670
13669
13667
13668
13698
28552
60
30
0
240
120
0
1.0
2.5
5.0
10
5
10
15
Time (minutes)
Time (minutes)
Intensity (A.U.)

Supplementary Figure S1: Comparing fluorescence intensity (arbitrary units) of loading observed in neuromasts and OHCs with derivatives of FM1-43 (groups I-VI) relative to the parent compound (13550). **(A)** (0.3 – 10 μM) shows results from zebrafish, with data points representing individual neuromasts from three independent replicates at each of four concentrations. **(B)** (0.3 μM only) shows results from mouse cochlear cultures and data points represent the mean from ten consecutive OHCs in an individual cochlear culture. Independent replicates and therefore number of datapoints are as follows: Zebrafish: FM1-43 (13550) n=8, 13695 n=3, 13696 n=3 14885 n=3, 16327 n=4, 14957 n=3, 13551 n=4, 13670 n=4, 13669 n=3, 13667 n=3, 13668 n=4, 13698 n=3 and 28552 n=3, Mouse: FM1-43 (13550) n=9, 13695 n=3, 13696 n=4, 14885 n=3, 16327 n=3, 14957 n=3, 13551 n=4, 13670 n=3, 13669 n=3, 13667 n=3, 13668 n=3, 13698 n=3 and 28552 n=3. Fitted lines (linear on a log-scale) show predicted responses to given treatment time (GLMM).

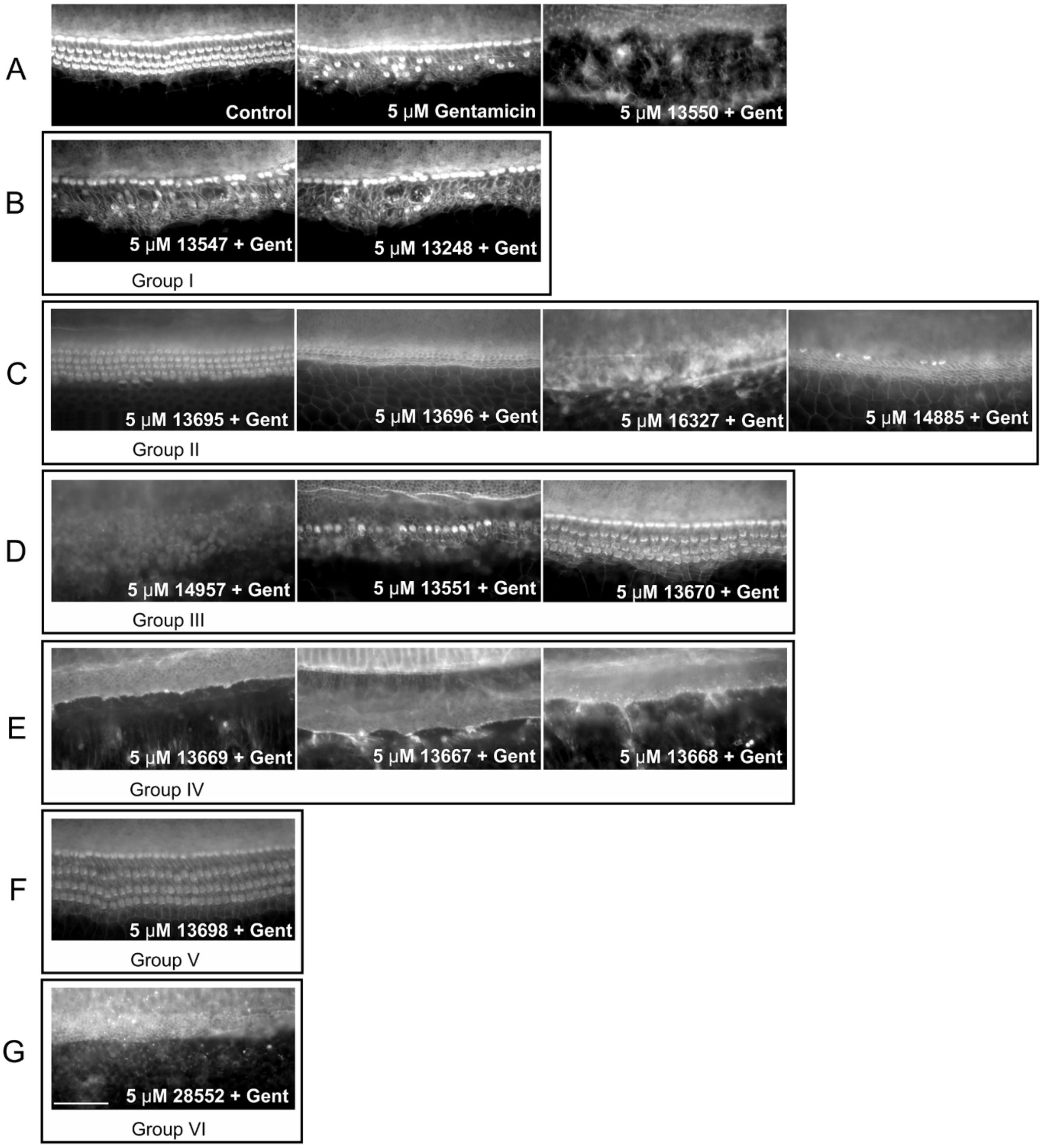

Supplementary Figure S2: Preliminary otoprotection screen with 5 µM FM1-43 and its derivatives. Cochlear cultures were treated with either 0.5% DMSO, 5 µM gentamicin + 0.5% DMSO, 5 µM gentamicin + 5 µM 13550 (parent compound) or 5 µM gentamicin + 5 µM of derivatives for 48 hours. (A) DMSO, 5 µM gentamicin, 13550, (B) truncated Group I structures; 13547 and 13248, (C) Group II compounds with lipophilic tail modifications; 13695, 13696, 16327 and 14885, (D) Group III compounds with hydrophilic head modifications; 14957, 1355 and 13670, (E) Group IV with linkers of varying length between the quaternary nitrogens; 13669, 13667 and 13668, (F) the Group V bis-derivative 13698, (G) the Group VI compound with modifications to both the lipophilic tail and hydrophilic head 28552. Scale bar is 50 µm.

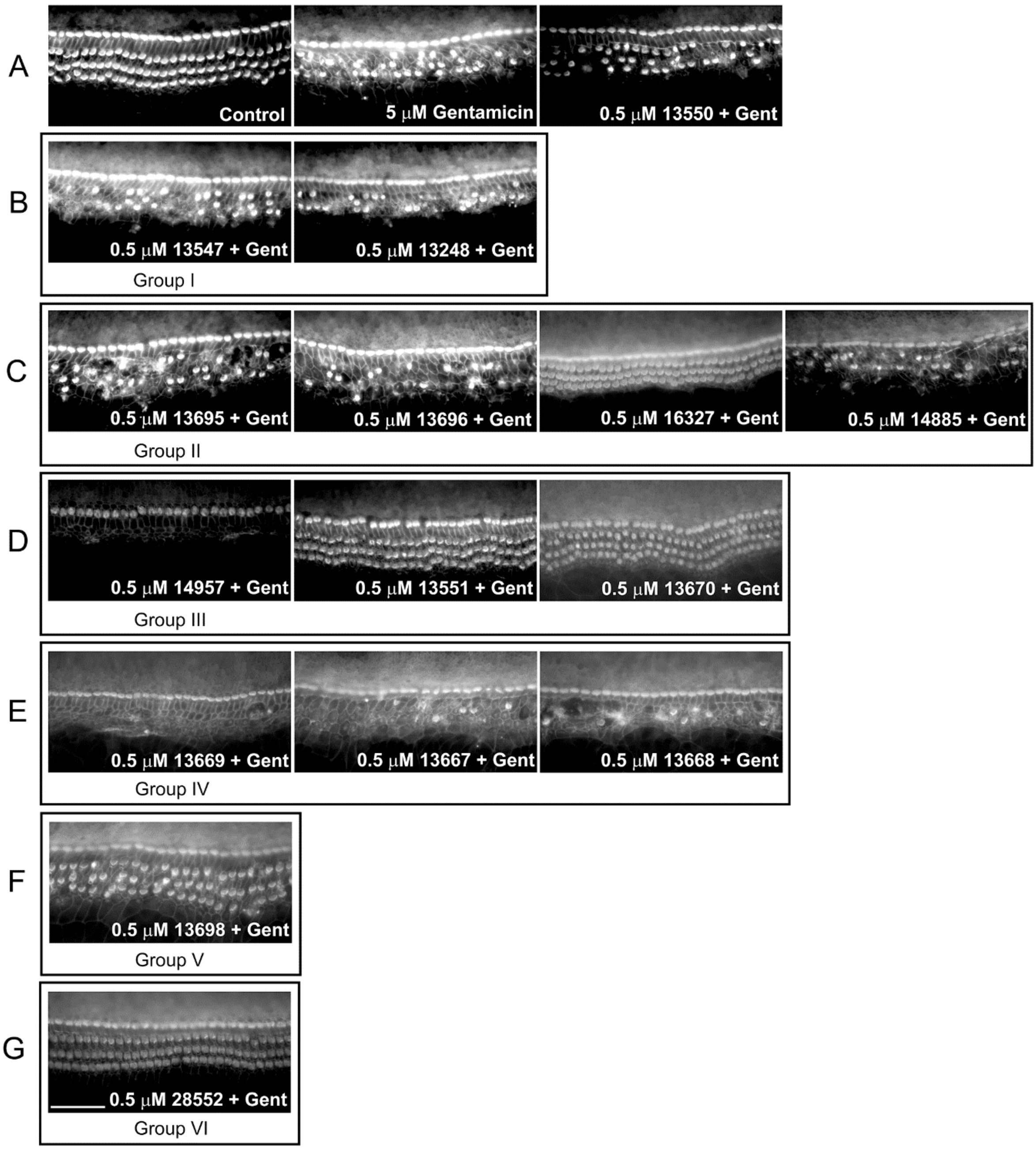

Supplementary Figure S3: Preliminary otoprotection screen with 0.5 μM FM1-43 and its derivatives. Cochlear cultures were treated with either 0.5% DMSO (control), 5 μM gentamicin + 0.5% DMSO, or 5 μM gentamicin + 0.5 μM of compound for 48 hours. (A) DMSO), 5 μM gentamicin, 13550. (B) truncated Group I compounds; 13547 and 13248, (C) Group II compounds with lipophilic tail modifications; 13695, 13696, 16327 and 14885, (D) Group III compounds with hydrophilic head modifications; 14957, 13551 and 13670, (E) Group IV compounds with linkers of varying length between the quaternary nitrogens; 13669, 13667 and 13668, (F) the Group V bis-derivative 13698 and (G) the Group IV compound with modifications to both the lipophilic tail and the hydrophilic head; 28552. Scale bar is 50 μm.

		Intercept				Concentration				Time			
		Est.	SE	z	p	Est.	SE	z	p	Est.	SE	z	p
I	*13547*	−1.324	0.072	−18.368	0.000	−0.384	0.045	−8.452	0.000	−0.117	0.068	−1.727	0.084
II	*13695*	−0.113	0.083	−1.363	0.173	−0.104	0.051	−2.044	0.041	−0.057	0.075	−0.769	0.442
	13696	−0.276	0.080	−3.438	0.001	−0.010	0.048	−0.203	0.839	−0.186	0.075	−2.479	0.013
	14885	−1.629	0.081	−20.027	0.000	−0.535	0.050	−10.750	0.000	−0.258	0.075	−3.438	0.001
	16327	−0.492	0.074	−6.668	0.000	−0.095	0.047	−2.037	0.042	−0.092	0.069	−1.349	0.177
III	*14957*	0.328	0.081	4.073	0.000	0.106	0.050	2.123	0.034	0.050	0.076	0.659	0.510
	13551	−0.501	0.072	−6.983	0.000	0.061	0.044	1.380	0.168	−0.041	0.069	−0.601	0.548
	13670	−1.441	0.071	−20.391	0.000	−0.359	0.043	−8.269	0.000	−0.159	0.068	−2.338	0.019
IV	*13669*	−0.616	0.084	−7.315	0.000	−0.204	0.051	−3.990	0.000	−0.070	0.076	−0.920	0.358
	13667	−0.666	0.083	−8.021	0.000	−0.155	0.049	−3.168	0.002	−0.081	0.076	−1.067	0.286
	13668	0.118	0.073	1.616	0.106	−0.072	0.046	−1.562	0.118	−0.048	0.068	−0.709	0.478
V	*13698*	−0.735	0.080	−9.171	0.000	−0.188	0.048	−3.887	0.000	−0.159	0.075	−2.110	0.035
VI	*28552*	−0.357	0.081	−4.412	0.000	0.024	0.052	0.461	0.645	−0.194	0.075	−2.566	0.010

Supplementary Table S1: Statistical analysis comparing fluorescence intensity (arbitrary units) in zebrafish neuromasts of derivatives of FM1-43 (groups I-VI) to the parent compound (13550). Est(imates) are the natural logarithm of the proportional change compared to 13550 (zero representing no proportional change), with their Standard Errors (SE), z-values, and p-values. **Intercept** is the comparison of 3 μM of each derivative with 3 μM of 13550 for 5 minutes (as main text Figure 7A). Derivatives that are significantly different to 13550 are highlighted in green. **Concentration** is the gradient of the fitted line (linear on a log-scale) through all concentrations tested for each derivative compared to the gradient for 13550. Derivatives that are significantly different to 13550 are highlighted in yellow. **Time** is the gradient of the line (linear on a log-scale) through all time points tested for each derivative compared to the 13550 gradient (GLMM). Derivatives that are significantly different to the 13550 are highlighted in purple. Red boxes surround those derivatives that were significantly brighter than 13550.

		Intercept				Time			
		Est.	SE	z	p	Est.	SE	z	p
II	*13695*	–0.073	0.123	–0.596	0.551	–0.026	0.019	–1.368	0.171
	13696	0.199	0.111	1.785	0.074	–0.011	0.017	–0.661	0.509
	14885	–1.226	0.123	–10.003	0.000	–0.057	0.019	–2.988	0.003
	16327	0.031	0.124	0.247	0.805	–0.011	0.019	–0.591	0.555
III	*14957*	1.182	0.124	9.544	0.000	–0.035	0.019	–1.815	0.070
	13551	0.358	0.111	3.242	0.001	–0.016	0.017	–0.919	0.358
	13670	–0.824	0.124	–6.672	0.000	–0.028	0.019	–1.450	0.147
IV	*13669*	0.391	0.122	3.199	0.001	–0.023	0.019	–1.234	0.217
	13667	–0.080	0.124	–0.645	0.519	–0.002	0.019	–0.088	0.930
	13668	0.269	0.124	2.160	0.031	0.017	0.019	0.871	0.384
V	*13698*	–0.710	0.123	–5.750	0.000	–0.031	0.019	–1.611	0.107
VI	*28552*	0.711	0.124	5.731	0.000	–0.032	0.019	–1.653	0.098

Supplementary Table S2: Statistical analysis comparing fluorescence intensity (arbitrary units) in mouse cochlear culture of derivatives of FM1-43 (groups I-VI) to the parent compound (13550). Est(imates) are the natural logarithm of the proportional change compared to 13550, with their Standard Errors (SE), z-values, and p-values are shown for: **Intercept** which is the comparison of 0.3 µM of each derivative with 0.3 µM of 13550 for 5 minutes (as main text Figure 7B). Derivatives that are significantly different to the 13550 are highlighted in green. **Time** which is the gradient of the line through all time points tested for each derivative compared to the 13550 gradient (GLMM). Derivatives that are significantly different to the 13550 highlighted in purple. Red boxes surround those derivatives that were significantly brighter than 13550.

A

	Est.	SE	z	p
13670	–0.119	0.101	–1.172	0.241
13695	–3.719	3.046	–1.221	0.222
13698	0.036	0.096	0.37	0.711
Gentamicin	–3.542	1.243	–2.849	0.004

B

	Est.	SE	z	p
13670	3.411	1.232	2.768	0.006
13695	–0.161	2.425	–0.067	0.947
13698	3.565	1.232	2.894	0.004
Control	3.529	1.231	2.868	0.004

C

	Est.	SE	z	p
13551	0.037	0.014	2.678	0.007
13670	–0.909	0.055	–16.554	0.001
16327	–0.526	0.054	–9.693	0.001
28552	0.009	0.012	0.767	0.443
Gentamicin	–4.216	1.03	–4.092	0.001

D

	Est.	SE	z	p
13551	4.329	0.609	7.102	0.001
13670	3.268	0.889	3.675	0.001
16327	3.751	0.64	5.863	0.001
28552	4.278	0.584	7.319	0.001
Control	4.279	0.607	7.045	0.001

Supplementary Table S3: Statistical analysis of Outer Hair Cell counts following treatment with derivatives of FM1-43. Estimated means (Est.) are the natural logarithm of the proportional difference compared to Controls (panels A and C) and Gentamicin alone (panels B and D) at treatment concentrations of 5 µM (panels A and B) and 0.5 µM (panels C and D), with their Standard Errors (SE). Z-values, and p-values are also shown from a Poisson GLMM.
